# Supplementary material for: Fast and selective organocatalytic ring-opening polymerization by fluorinated alcohol without a cocatalyst
Source: Nat Commun. 2019 Aug 9;10:3590. doi: 10.1038/s41467-019-11524-y (PMC6689068; doi:10.1038/s41467-019-11524-y)
Supplement: Supplementary file 1 — Supplementary Information [file 41467_2019_11524_MOESM1_ESM.pdf]

Supplementary Information

Fast and Selective Organocatalytic Ring-Opening Polymerization by Fluorinated Alcohol without a Cocatalyst

Zhao *et al.*

Supplementary Methods

Materials. All reactions were carried out under a dry and oxygen-free argon atmosphere by using Schlenk techniques or under an argon atmosphere in an MBraun glovebox. Solvents such as dichloromethane (DCM, ≥99.5%, Sigma-Aldrich) and tetrahydrofuran (THF, ≥99.0%, Sigma-Aldrich) were purified using an MBraun SPS system. DCM used for polymerization was further dried by passing through an activated alumina column. THF was further dried with sodium-potassium alloy until a characteristic blue color was evident in the solvent. Deuterated dichloromethane (DCM-d_2, 99.9 atom % D, Sigma-Aldrich) used for NMR experiments was dried over phosphorus pentoxide (P_2O_5, 99.0%, Sigma-Aldrich) at room temperature under Ar atmosphere overnight followed by distillation under reduced pressure. All other liquids including 2-(Dimethylamino)-ethanol (DMEA, ≥99.5%, Sigma-Aldrich) and 1,3-Bis(hexafluoro-α-hydroxyisopropyl)benzene (1,3-Bis-HFAB, ≥98%, Sigma-Aldrich) were dried over activated 4 Å molecular sieves for a week and distilled before use, and solid materials were used as received. All purified reagents were stored over 4Å molecular sieves in a glove box. H-Glu(OBn)-OH (L-Glutamic acid *γ*-benzyl ester, 98%), H-Lys(Z)-OH (*N*ε-carbobenzoxy(CBZ)-L-lysine, 98%) propargyl alcohol (98%), trimethylsilyl chloride (99%) and *t*-Butyldimethylchlorosilane (99%) were purchased from Sigma-Aldrich and used as received. Glu-NCA and Lys-NCA were prepared and recrystallized four times according to published procedures.[1] Alkynyl-NCA was prepared and purified according to the literature.[2] 2-(tert-butyldimethylsilyloxy)-N,N-dimethylethanamine (DMEA-TMS') was prepared and purified according to the literature.[3]

Density Functional Theory (DFT) Calculation Details. The calculation was performed at the GGA/PBE/DNP level of theory. All of the constructed structures are fully optimized and electrostatic potential (ESP)-fitting charges are derived from the DFT calculation. (Frisch, M. J.; Trucks, G. W.; Schlegel, H. B.; Scuseria, G. E.; Robb, M. A.; Cheeseman, J. R.; Scalmani, G.; Barone, V.; Mennucci, B.; Petersson, G. A.; Nakatsuji, H.; Caricato, M.; Li, X.; Hratchian, H. P.; Izmaylov, A. F.; Bloino, J.; Zheng, G.; Sonnenberg, J. L.; Hada, M.; Ehara, M.; Toyota, K.; Fukuda, R.; Hasegawa, J.; Ishida, M.; Nakajima, T.; Honda, Y.; Kitao, O.; Nakai, H.; Vreven, T.; Montgomery, Jr., J. A.; Peralta, J. E.; Ogliaro, F. O.; Bearpark, M. J.; Heyd, J.; Brothers, E. N.; Kudin, K. N.; Staroverov, V.

N.; Kobayashi, R.; Normand, J.; Raghavachari, K.; Rendell, A. P.; Burant, J. C.; Iyengar, S. S.; Tomasi, J.; Cossi, M.; Rega, N.; Millam, N. J.; Klene, M.; Knox, J. E.; Cross, J. B.; Bakken, V.; Adamo, C.; Jaramillo, J.; Gomperts, R.; Stratmann, R. E.; Yazyev, O.; Austin, A. J.; Cammi, R.; Pomelli, C.; Ochterski, J. W.; Martin, R. L.; Morokuma, K.; Zakrzewski, V. G.; Voth, G. A.; Salvador, P.; Dannenberg, J. J.; Dapprich, S.; Daniels, A. D.; Farkas, D. N.; Foresman, J. B.; Ortiz, J. V.; Cioslowski, J.; Fox, D. J. Gaussian 09; Gaussian, Inc.: Wallingford, CT, 2009.)

Supplementary Discussion

Competition of two different mechanisms in the ROP of α-amino acid NCAs. The chemistry of NCAs is unique: the carbonyl group (C5) of NCA is highly electrophilic and can be readily attacked by various nucleophiles, inducing ROP (via the "normal amine mechanism", Supplementary Figure 1, Path 1). Furthermore, the acidic proton at the N3 position can be readily deprotonated under basic conditions. The resultant NCA nucleophilic anion can attack the carbonyl group (C5) of another NCA molecule, triggering an uncontrolled ROP of NCA, the so-called "activated monomer mechanism" (AMM, Supplementary Figure 1, Path 2), or can undergo rearrangement to form isocyanocarboxylates, which terminate the growing chains. The "normal amine" (NAM) and "activated monomer" (AMM) mechanisms, often compete each other and complicate the overall polymerization process, making it challenging to produce well-defined (co)polypeptides.[4,5]

ROP of α-amino acid NCAs catalyzed by Hexafluoroisopropanol (HFIP). HFIP was also used a potential catalyst instead of 1,3-bis-HFAB for ROP of α-amino acid NCAs. (polymerization conditions: [Glu-NCA]/[DMEA]/[HFIP]=120/1/1, [Glu-NCA]=0.19M, 25°C). As expected, the results are not as good as those using 1,3-bis-HFAB. The resultant polypeptide has a bimodal molecular weight distribution, indicating that HFIP may participate in the initiation (presumably because of lower steric hindrance) and/or has no capability to silent the initiating action of tertiary amine in DMEA. We monitored the NCA polymerization with HFIP by using in situ FT-IR (polymerization conditions: [Glu-NCA]/[HFIP]=60, [Glu-NCA]=0.19M, 25°C) and found that HFIP initiated the NCA polymerization in a slow and uncontrolled manner (4.4h, 85% conversion, $M_{n,SEC}$=3.44×10^{4}, Đ=1.22, Supplementary Figure 24). We also studied the NCA polymerization by

using silyl-protected ethanolamine (DMEA-TMS')/HFIP (polymerization conditions: [Glu-NCA]/[DMEA-TMS']/[HFIP]=120/1/1, [Glu-NCA]=0.19M, 25°C). The polymerization is slow (3h, 93% conversion) and the resultant polypeptide has a much higher molecular weight than the theoretical one and a bimodal molecular weight distribution ($M_{n,SEC}$=2.67×10^{5}, Đ=1.63, Supplementary Figure 25). This is because HFIP participates in the initiation and cannot silent the tertiary amine in DMEA, so both the NAM and AMM pathways co-exist in the polymerization system. In contrast, for 1,3-bis-HFAB used in this study, the bulky electron-withdrawing fluorinated groups can increase the acidity of the alcohol (increasing hydrogen bonding), while steric factors reduce the nucleophilicity of the alcohols and prevent its participation in initiation. That is why 1,3-bis-HFAB shows much better results than HFIP.

ROP of α-amino acid NCAs initiated by triethylamine (TEA). A control experiment by using tertiary amine, triethylamine (TEA), as a potential initiator in the absence of 1,3-bis-HFAB was carried out (conditions: [Glu-NCA]/[TEA]=120, $[NCA]_0$=0.19M, 25°C, CH_2Cl_2). The results showed that the polymerizations were very slow (24h, 90% conv.) and uncontrollable, resulting in polypeptides with very broad unsymmetrical SEC trace (Đ=1.80) and much higher molecular weight ($M_{n,SEC}$=3.92×10^{5}) than theoretical one ($M_{n,calcd}$ =2.37×10^{4}) (Supplementary Figure 26). The $M_{n,SEC\text{-}MALS}$ is 16 times higher than that of the calculated molecular weight. The kinetics study showed that a first-order dependence of polymerization rate on monomer concentration was not observed, indicating slow initiating of the polymerization.

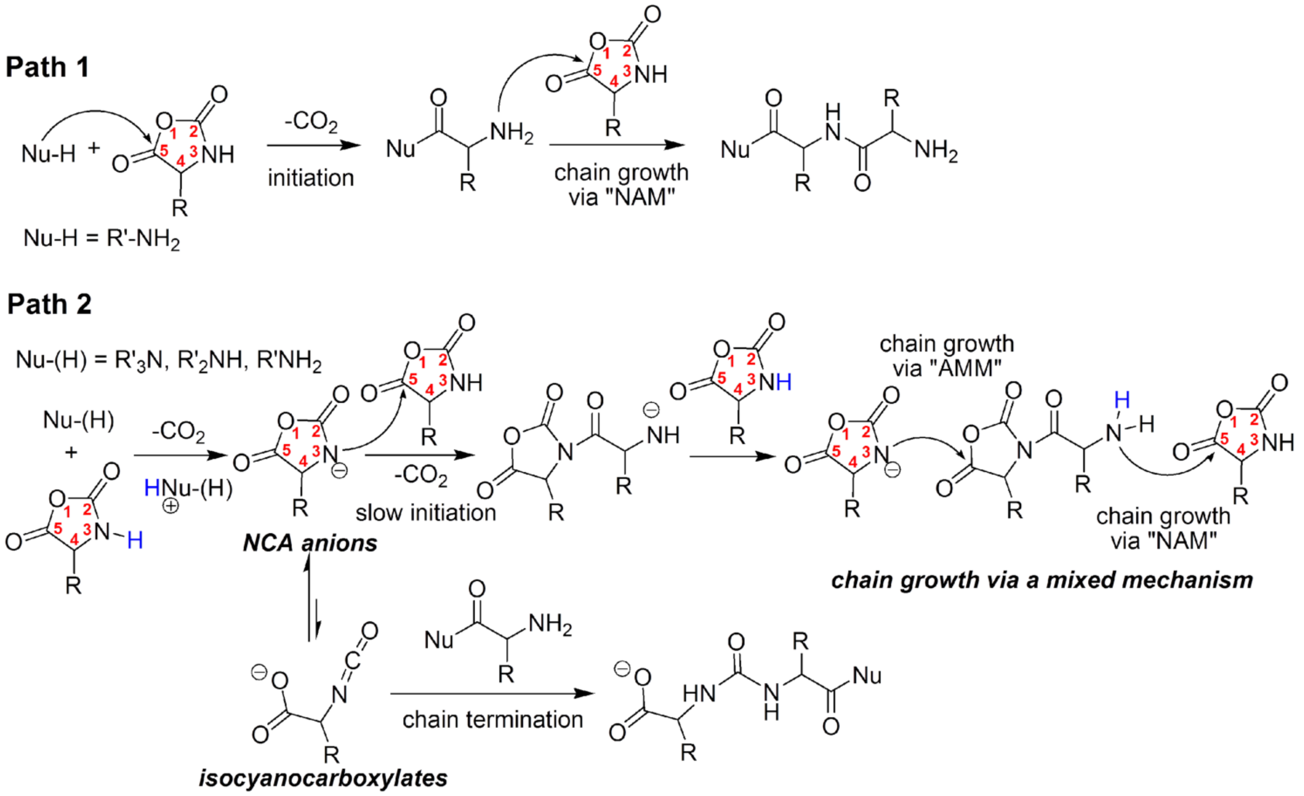

Supplementary Figure 1 Competition of the "normal amine mechanism" (NAM, Path 1) and "activated monomer mechanism" (AMM, Path 2) in the ROP of α-amino acid NCAs

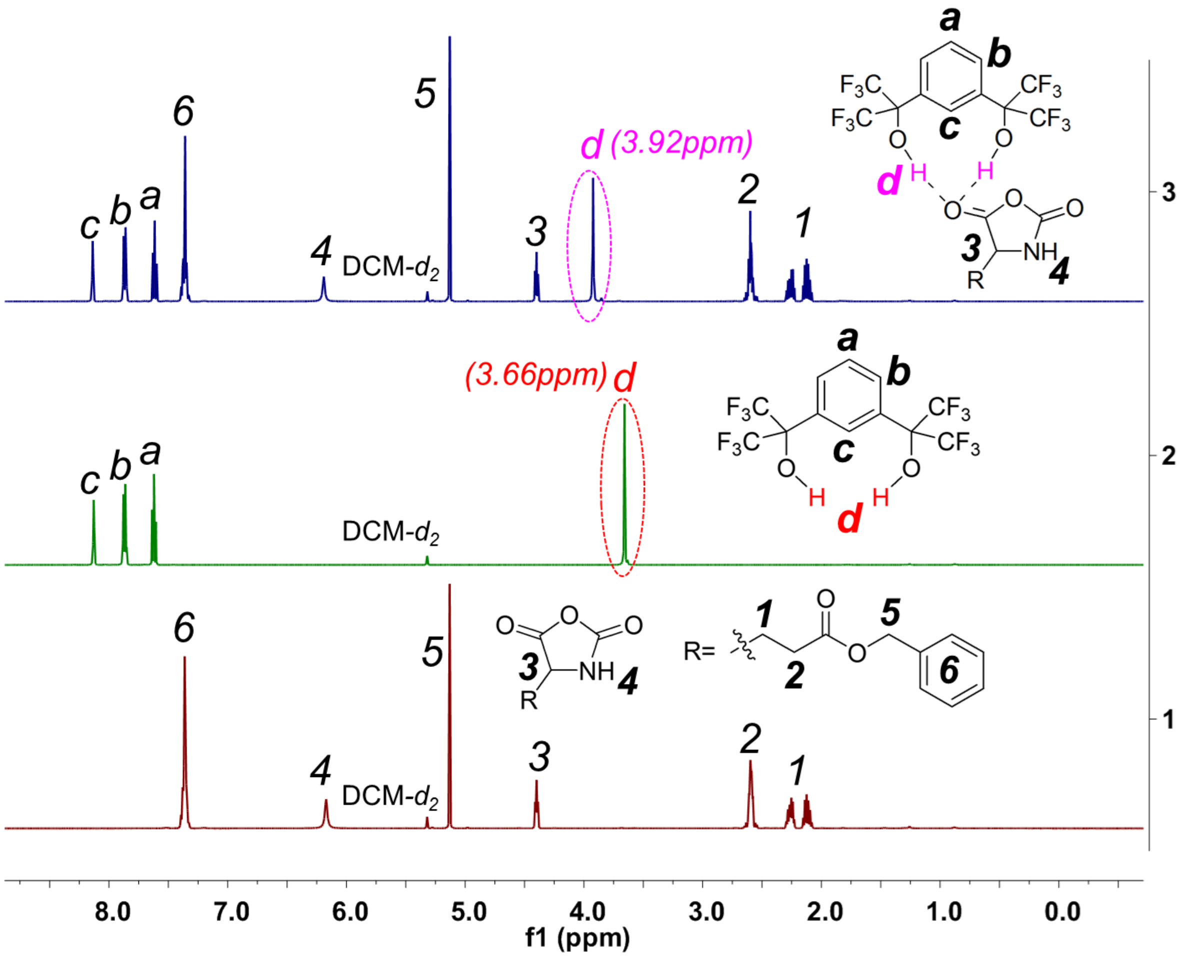

Supplementary Figure 2 Full ^{1}H NMR spectra of 1,3-Bis-HFAB, Glu-NCA and 1,3-Bis-HFAB/Glu-NCA (1:1) mixture (500M, 25°C, CD_2Cl_2) (Source data are provided as a Source Data file)

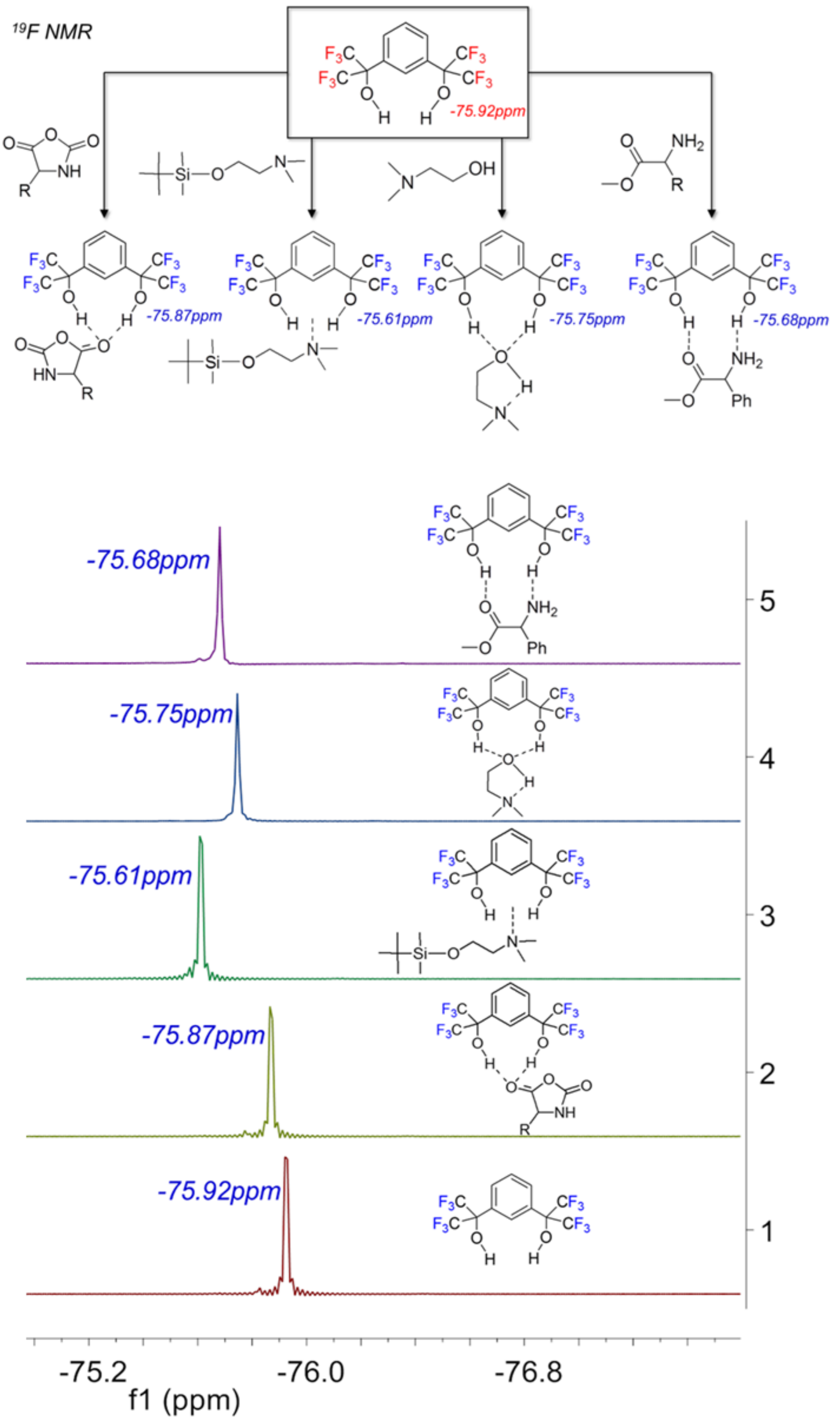

Supplementary Figure 3 Full ^{19}F NMR spectra of 1,3-Bis-HFAB, 1,3-Bis-HFAB/Glu-NCA (1:1), 1,3-Bis-HFAB/DMEA-TMS’ (1:1), 1,3-Bis-HFAB/DMEA (1:1) and 1,3-Bis-HFAB/ MAP (1:1) mixtures (500M, 25°C, CD_2Cl_2) (Source data are provided as a Source Data file)

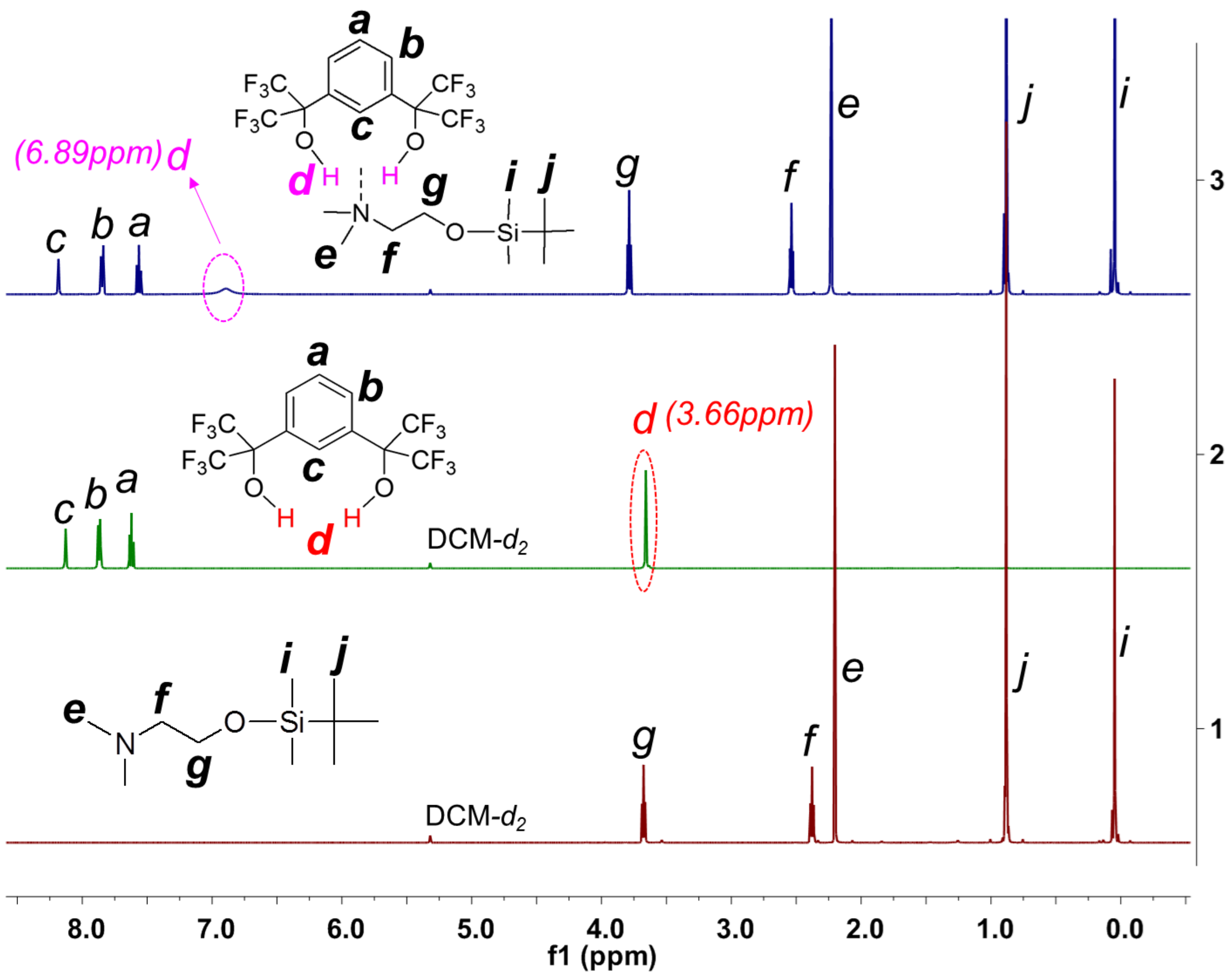

Supplementary Figure 4 Full ^{1}H NMR spectra of 1,3-Bis-HFAB, DMEA-TMS' and 1,3-Bis-HFAB/DMEA-TMS' (1:1) mixture (500M, 25°C, CD_2Cl_2) (Source data are provided as a Source Data file)

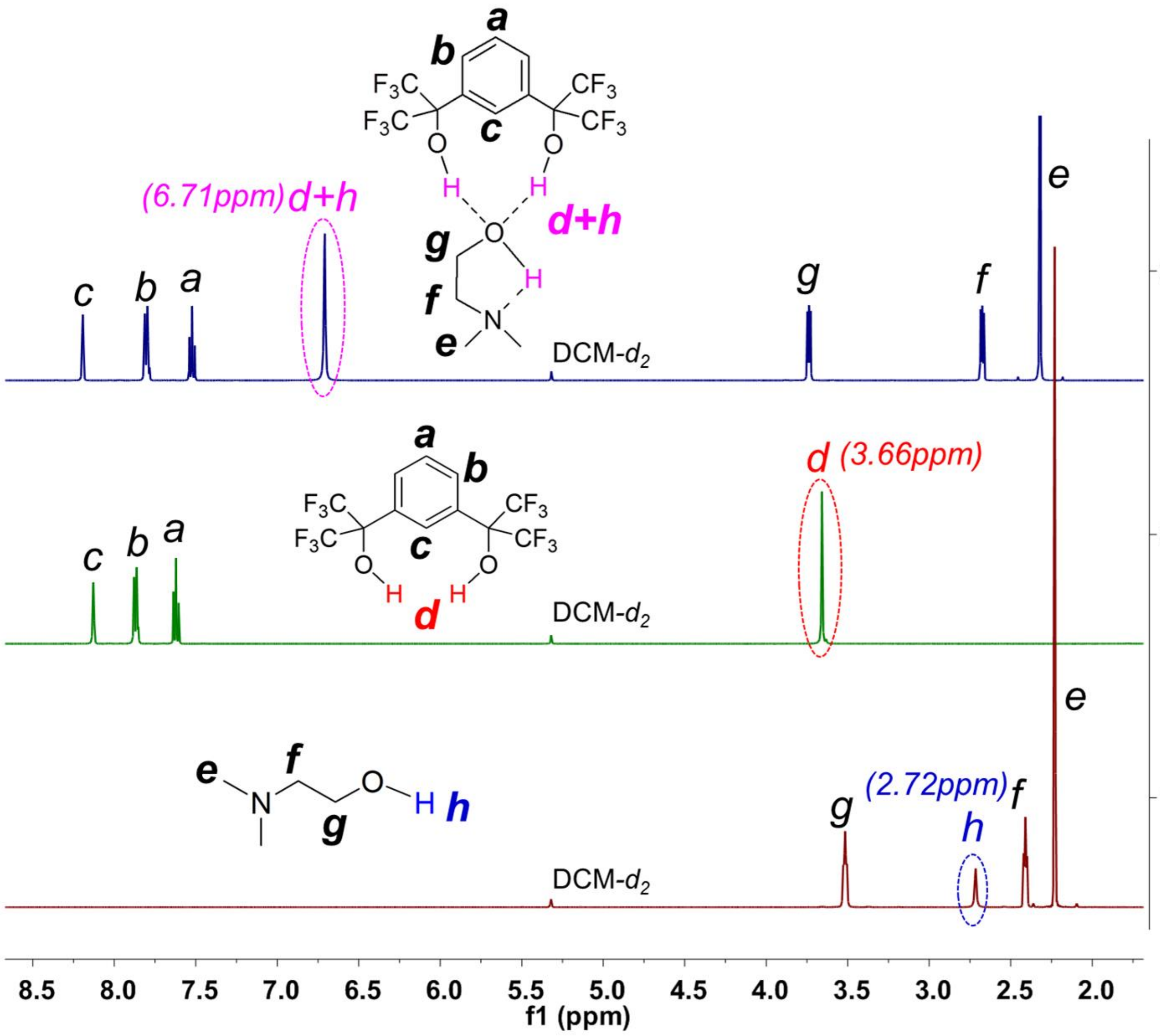

Supplementary Figure 5 Full ^{1}H NMR spectra of 1,3-Bis-HFAB, DMEA and 1,3-Bis-HFAB/DMEA (1:1) mixture (500M, 25°C, CD_2Cl_2) (Source data are provided as a Source Data file)

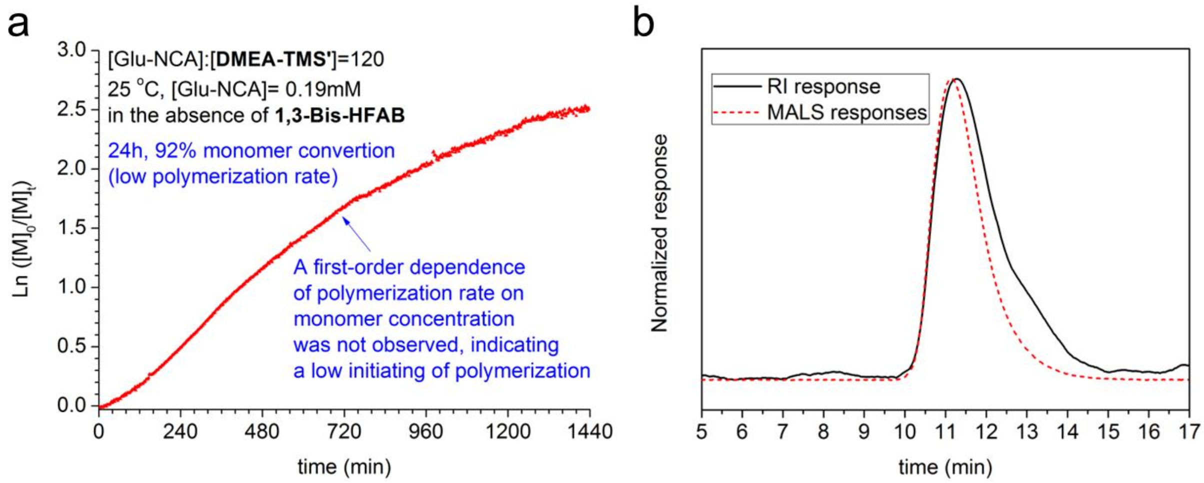

Supplementary Figure 6 Kinetics study of ROP of Glu-NCA initiated by DMES-TMS' in the absence of 1,3-bis-HFAB. (a) plot of Ln ($[M]_0/[M]_t$) vs. time, conditions: [Glu-NCA]/[DMES-TMS']=120, $[NCA]_0$=0.19M, 25°C, CH_2Cl_2; (b) corresponding SEC traces of obtained polypeptide ($M_{n,SEC}=2.67\times10^5$, Đ=1.63); Note: the $M_{n,SEC}$ is ten times that of the calculated molecular weight ($M_{n,calcd}=2.63\times10^4$), indicating the polymerization proceeds through activated monomer mechanism) (Source data are provided as a Source Data file)

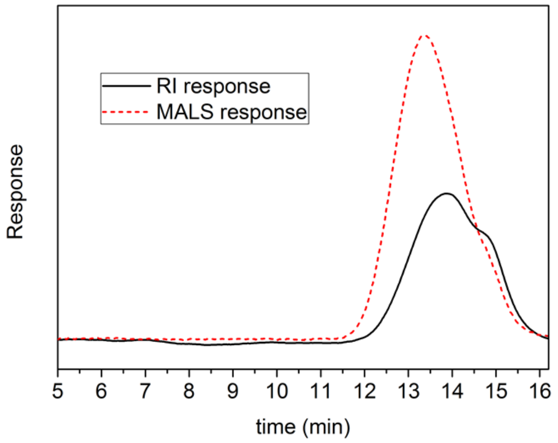

Supplementary Figure 7 SEC traces of polypeptide prepared from ROP of Glu-NCA initiated by DMEDA (conditions: [Glu-NCA]/[DMEDA]=100, [Glu-NCA]=0.19M, 25°C) (Source data are provided as a Source Data file)

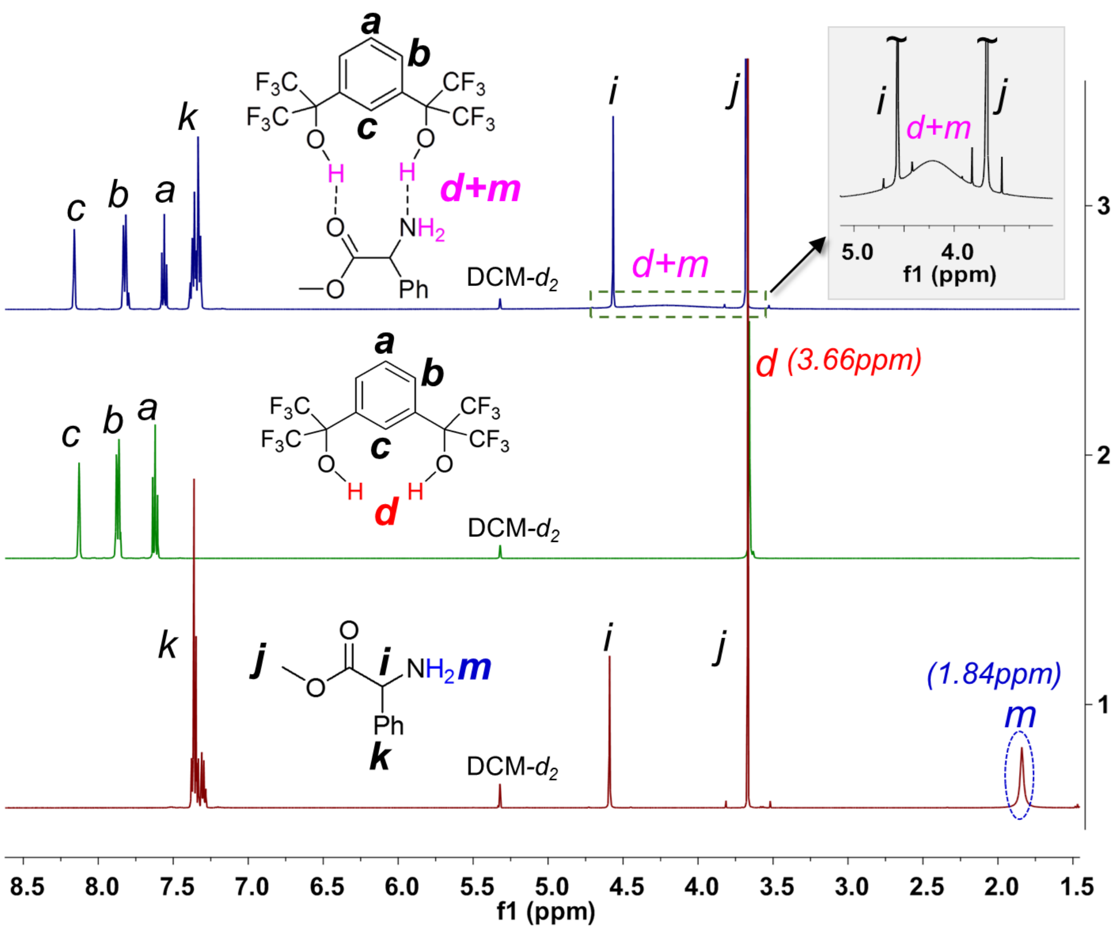

Supplementary Figure 8 Full ^{1}H NMR spectra of 1,3-Bis-HFAB, MAP and 1,3-Bis-HFAB/ MAP (1:1) mixture (500M, 25°C, CD_2Cl_2) (Source data are provided as a Source Data file)

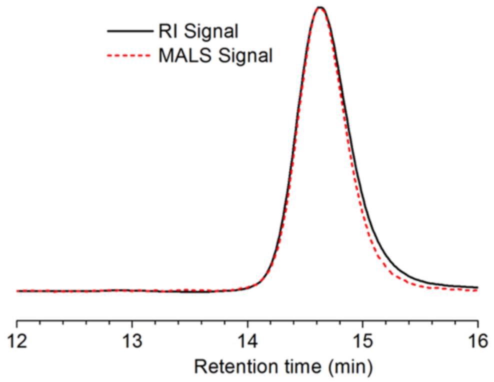

Supplementary Figure 9 SEC traces of polypeptide from ROP of Glu-NCA catalyzed by 1,3-Bis-HFAB ($[Glu\text{-}NCA]_0$ = 0.19 M, [Glu-NCA]/[1,3-Bis-HFAB]/[DMEA] = 120/(10+30)/1, 25 ℃, CH_2Cl_2, 30 *eq*. 1,3-Bis-HFAB was added into system during polymerization, solid lines: RI response, dash lines: MALS response) (Source data are provided as a Source Data file)

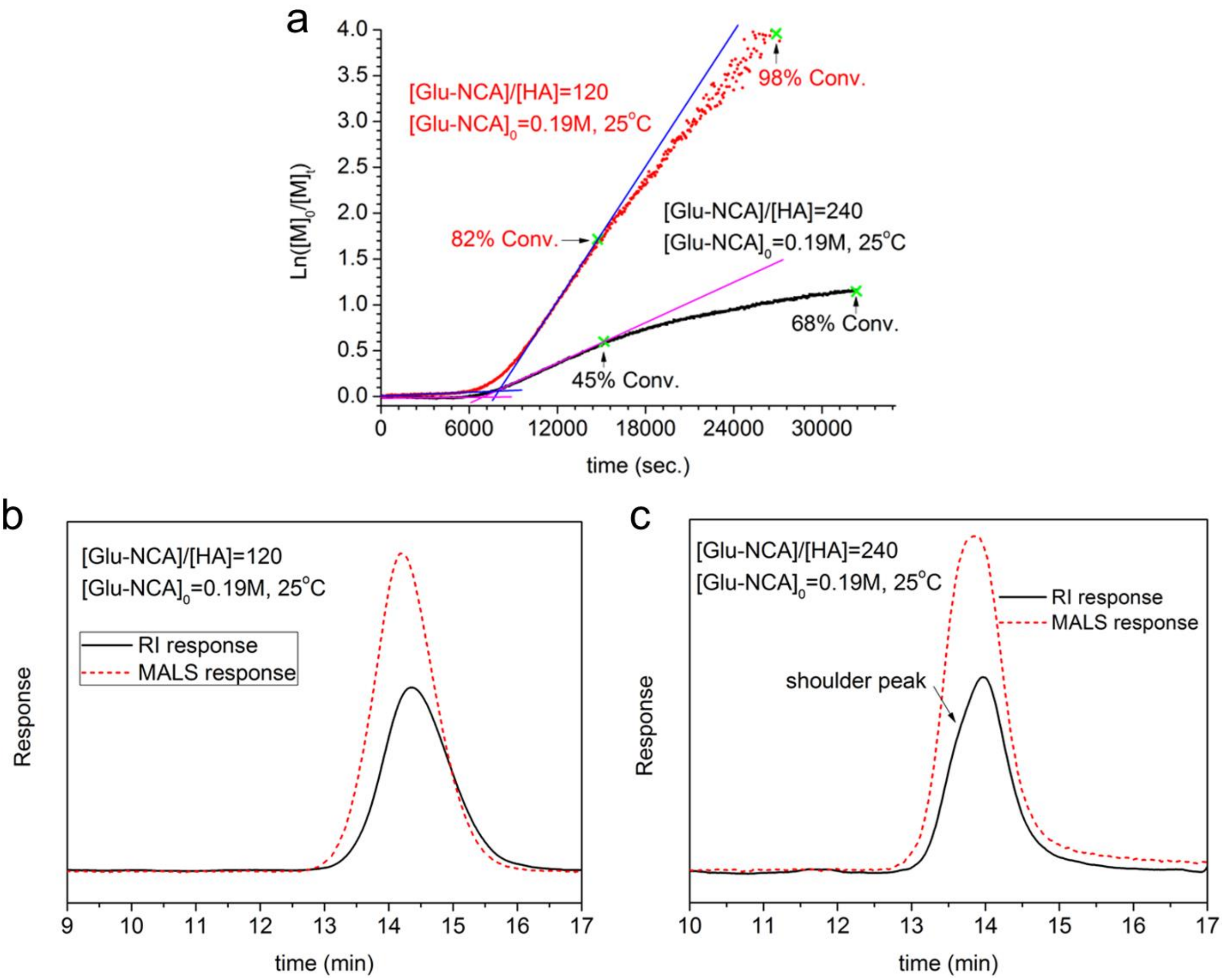

Supplementary Figure 10 Kinetics study of ROP of NCA initiated by n-hexylamine (HA). (a) plots of Ln($[M]_0/[M]_t$ vs. time; (b) and (c) corresponding SEC traces of resultant polypeptides (polymerization conditions: [Glu-NCA]/[n-hexylamine]=120 and 240, [Glu-NCA]=0.19M, 25°C, CH_2Cl_2). Note: When the monomer/initiator ratio was increased from 120 to 240, a shoulder peak was observed in the SEC trace of resultant polypeptide and the $M_{n,SEC-MALS}$ is nearly two times that of $M_{n,calcd}$, indicating a poor control of the polymerization (Source data are provided as a Source Data file)

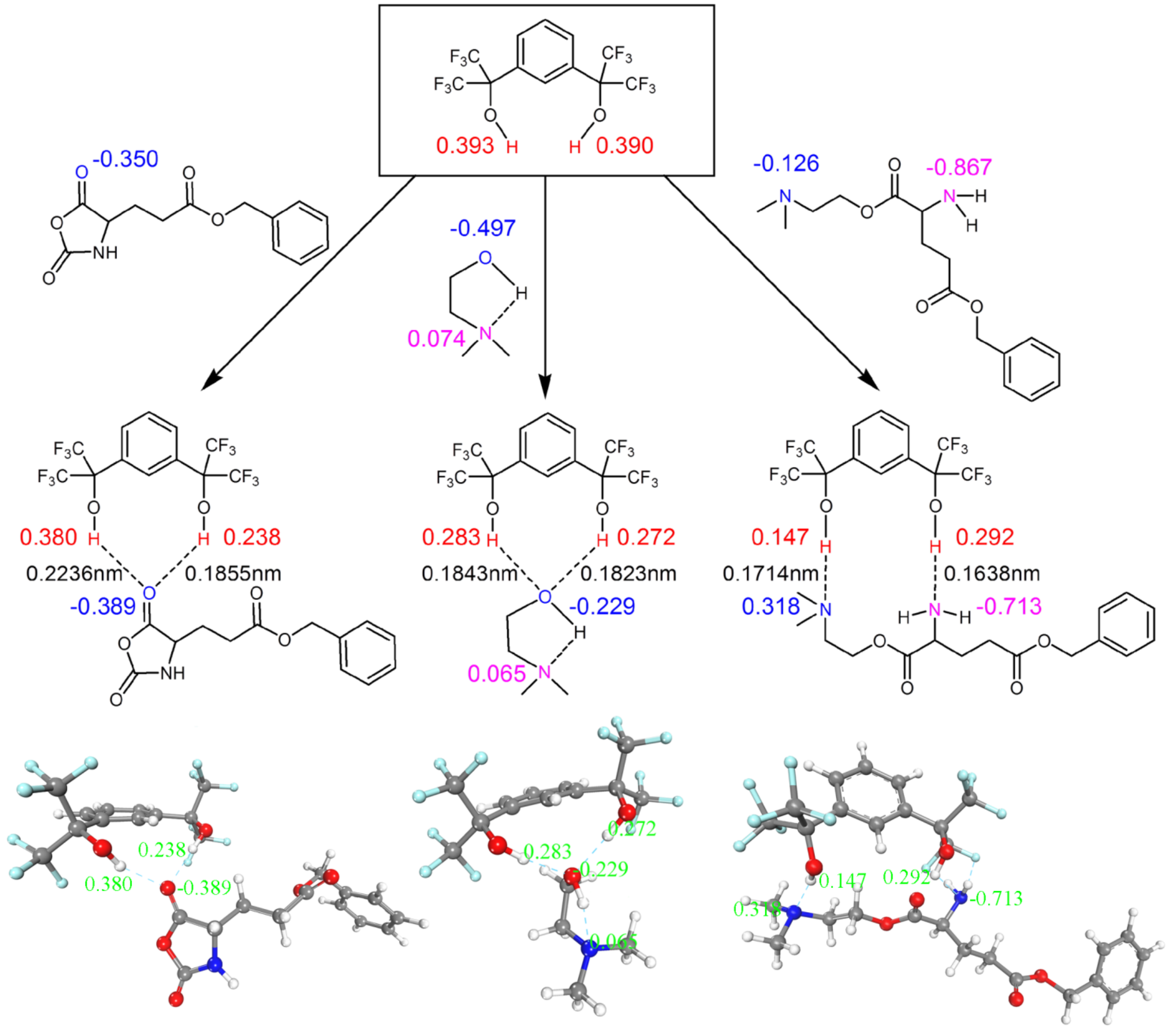

Supplementary Figure 11 ESP charge changes of selected atoms in the lowest-energy structures of complexes 1,3-Bis-HFAB/Glu-NCA, 1,3-Bis-HFAB/DMEA and 1,3-Bis-HFAB/(DMEA+one inserted Glu-NCA). (Structures of alternative, higher-energy complexes are listed in supplementary Table 1)

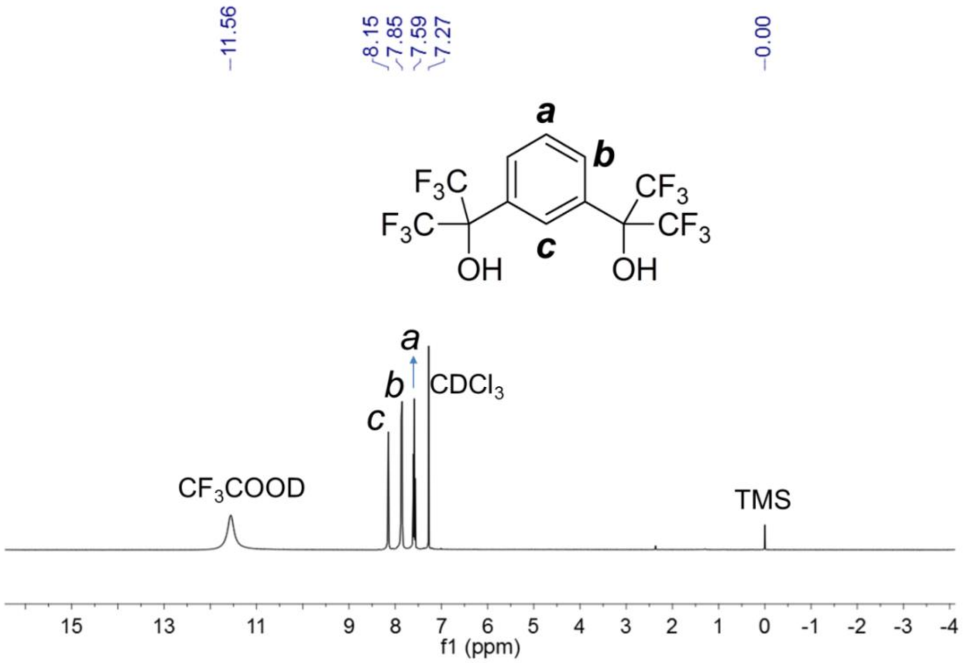

Supplementary Figure 12 ^{1}H NMR spectrum of 1,3-Bis-HFAB (500M, 25°C, $CDCl_3/CF_3COOD$ (2:1)) (Source data are provided as a Source Data file)

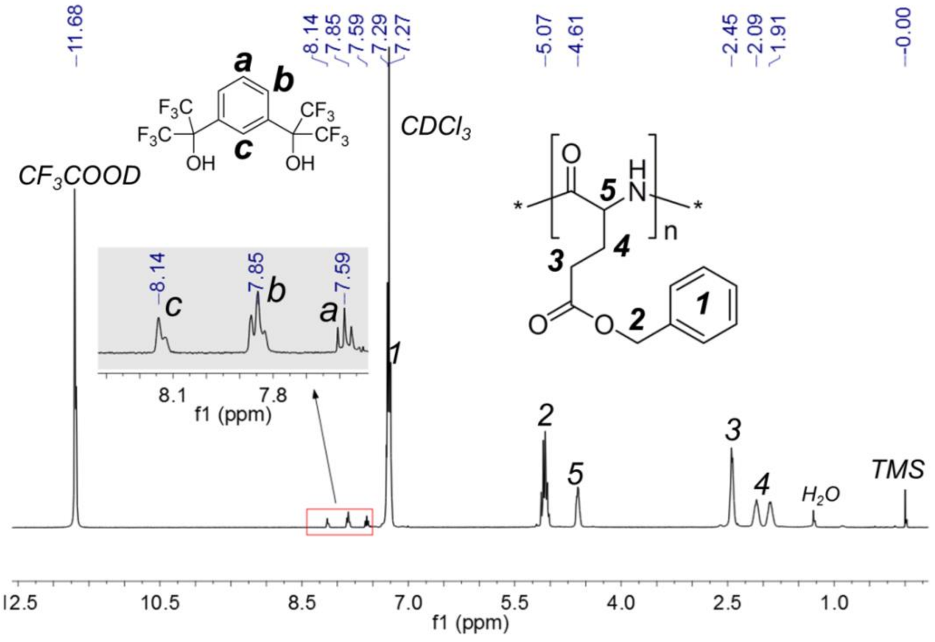

Supplementary Figure 13 ^{1}H NMR spectrum of PBLG from polymerization initiated by DMEA/1,3-Bis-HFAB (500M, 25°C, $CDCl_3/CF_3COOD$ (2:1), before precipitation with methanol) (Source data are provided as a Source Data file)

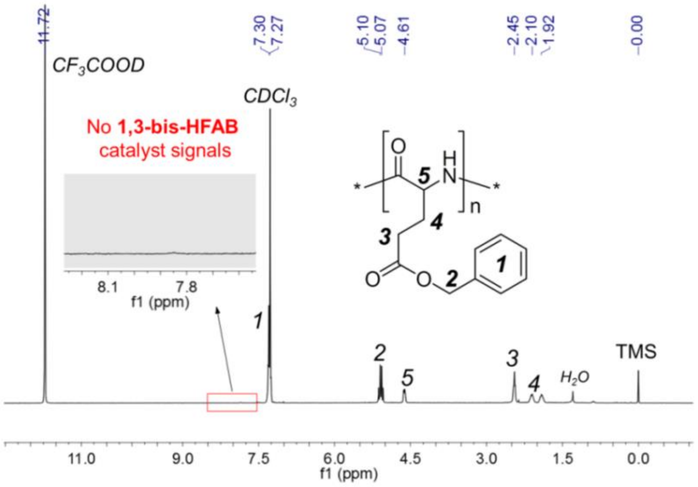

Supplementary Figure 14 ^{1}H NMR spectrum of PBLG from polymerization initiated by DMEA/1,3-Bis-HFAB (500M, 25°C, $CDCl_3/CF_3COOD$ (2:1), After precipitation with methanol, the resultant PBLG was further washed with methanol two times) (Source data are provided as a Source Data file)

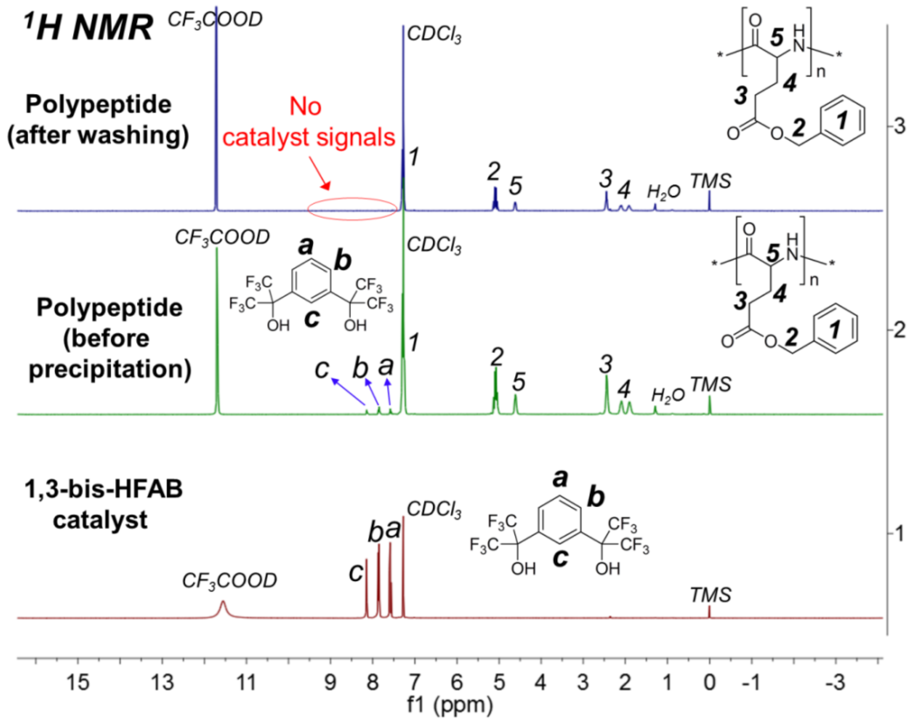

Supplementary Figure 15 The combined ^{1}H NMR spectra of 1,3-bis-HFAB catalyst and PBLG from polymerization initiated by DMEA/1,3-Bis-HFAB (500M, 25°C, $CDCl_3/CF_3COOD$ (2:1). (1) 1,3-bis-HFAB catalyst; (2) before precipitation with methanol; (3) the precipitated PBLG was further washed with methanol twice) (Source data are provided as a Source Data file)

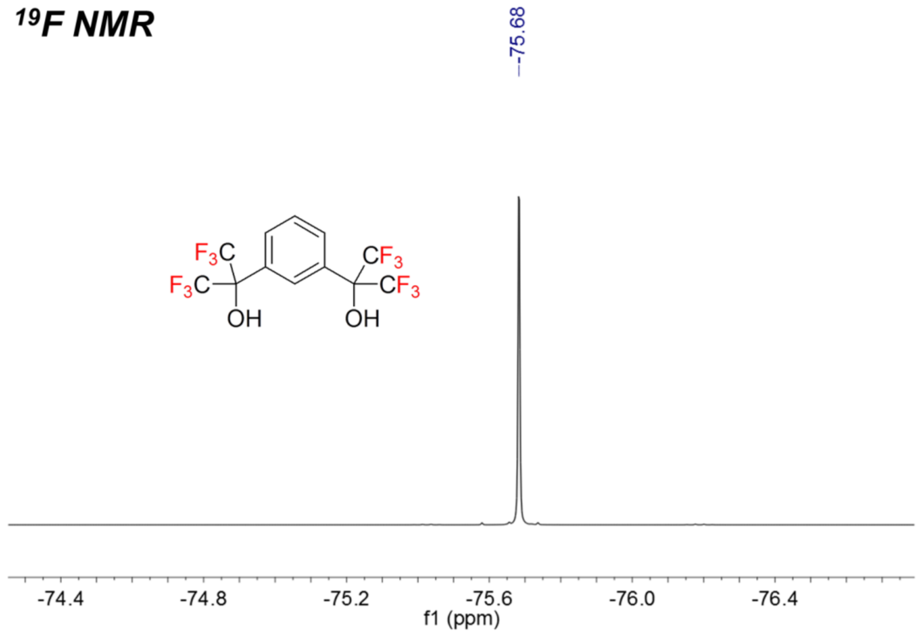

Supplementary Figure 16 ^{19}F NMR spectrum of 1,3-Bis-HFAB (500M, 25°C, $CDCl_3$) (Source data are provided as a Source Data file)

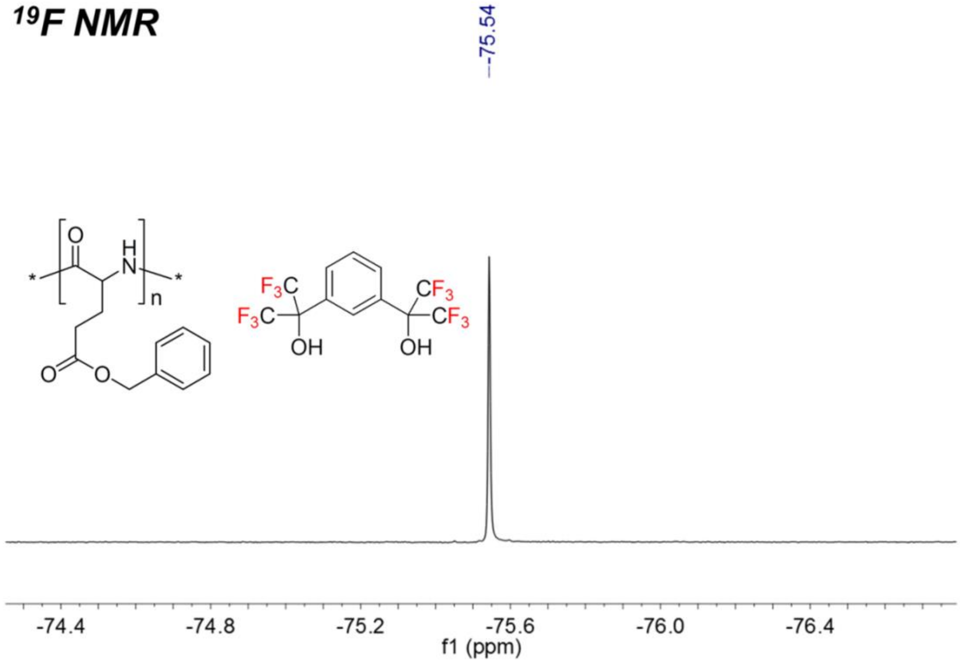

Supplementary Figure 17 ^{19}F NMR spectrum of PBLG from polymerization initiated by DMEA/1,3-Bis-HFAB (500M, 25°C, $CDCl_3$, before precipitation with methanol) (Source data are provided as a Source Data file)

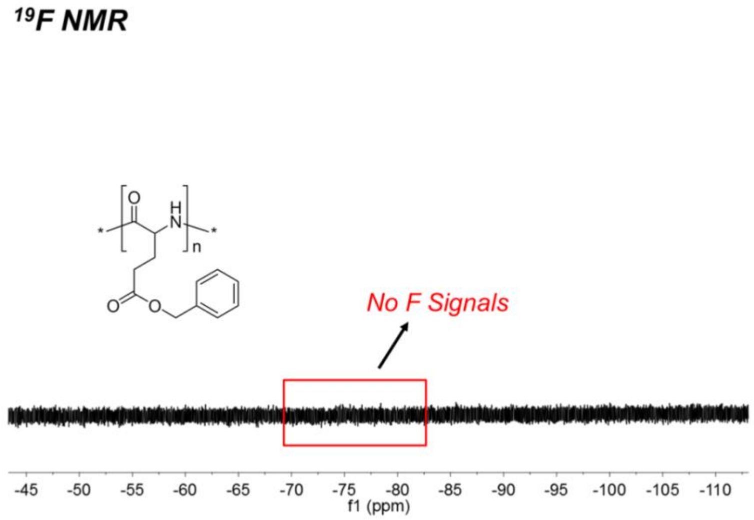

Supplementary Figure 18 ^{19}F NMR spectrum of PBLG from polymerization initiated by DMEA/1,3-Bis-HFAB (500M, 25°C, $CDCl_3$, the precipitated PBLG was further washed with methanol twice) (Source data are provided as a Source Data file)

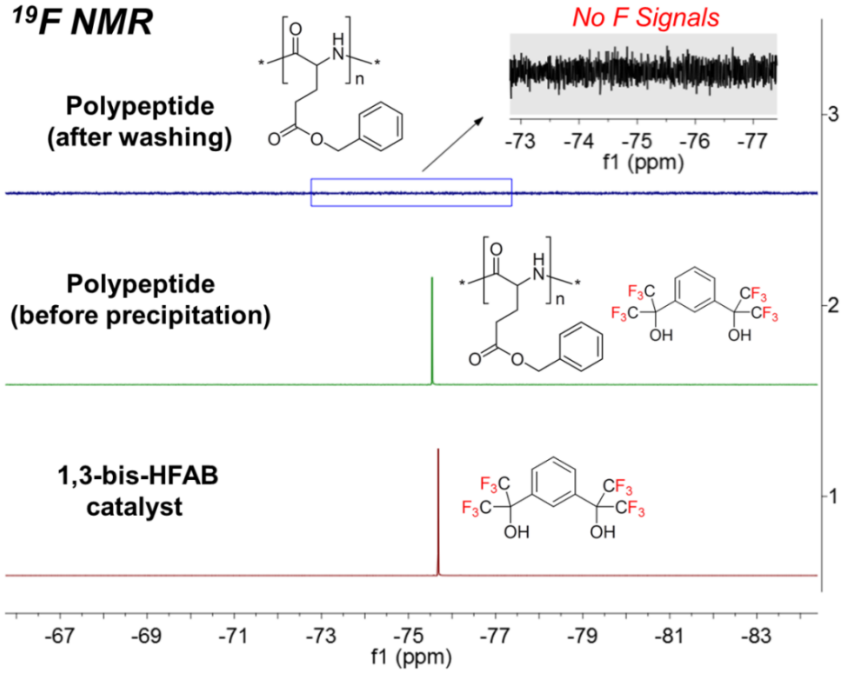

Supplementary Figure 19 The combined ^{19}F NMR spectra of 1,3-bis-HFAB catalyst and PBLG from polymerization initiated by DMEA/1,3-Bis-HFAB (500M, 25°C, $CDCl_3$). (1) 1,3-bis-HFAB catalyst; (2) before precipitation with methanol; (3) the precipitated PBLG was further washed with methanol twice)

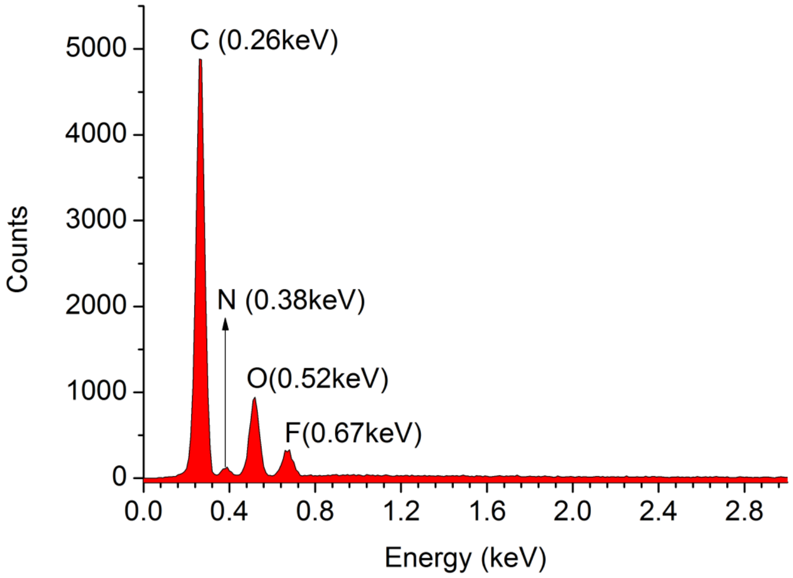

Supplementary Figure 20 EDS spectrum of PBLG from polymerization initiated by DMEA/1,3-Bis-HFAB (before precipitation with methanol) (Source data are provided as a Source Data file)

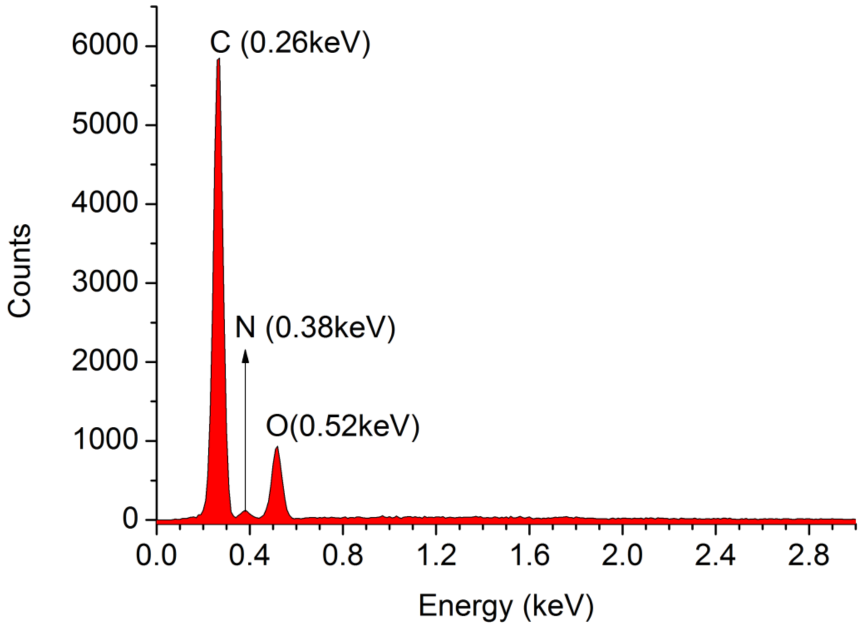

Supplementary Figure 21 EDS spectrum of PBLG from polymerization initiated by DMEA/1,3-Bis-HFAB (the precipitated PBLG was further washed with methanol twice) (Source data are provided as a Source Data file)

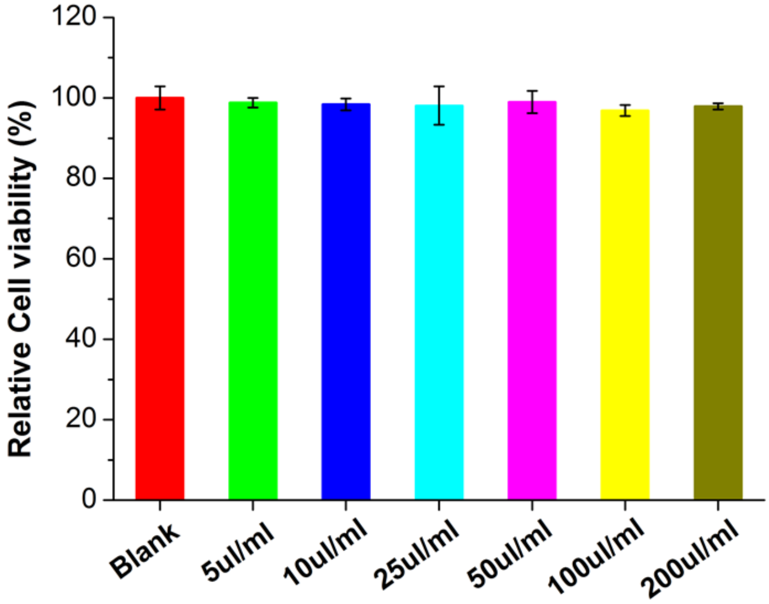

Supplementary Figure 22 In vitro cytotoxicity evaluation of the final polypeptide by the viability of HepG2 cells cultured for 48 h with various concentrations of the extract from the final polypeptides(Source data are provided as a Source Data file)

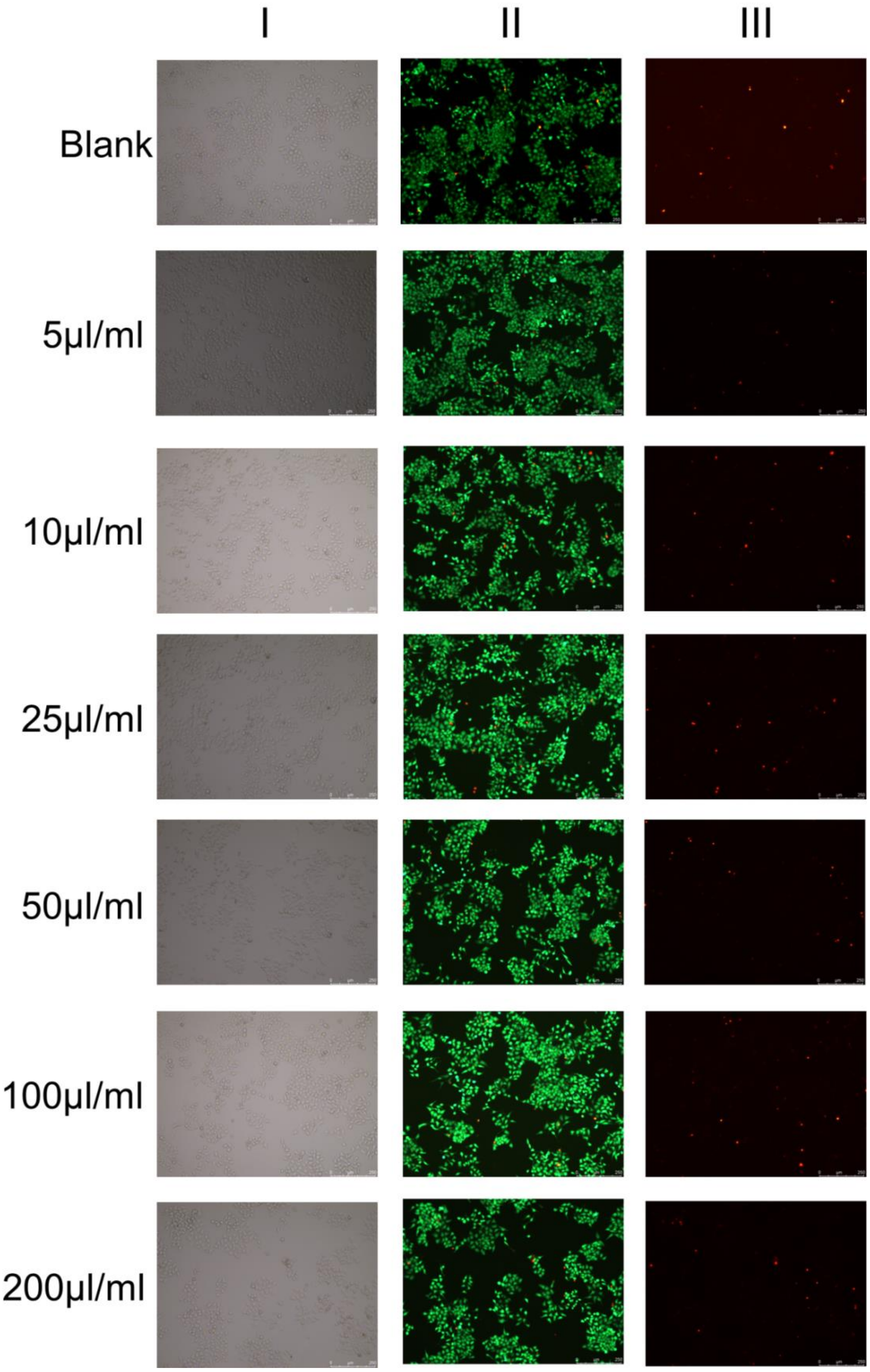

Supplementary Figure 23 Fluorescence microscopy images of HepG2 cells cultured for 48 h with various concentrations of the extract from the final polypeptides. (I) in bright-field; (II) images of cells observed in dark-field with $\lambda_{excitation}$=488nm, green color represents living cell and red for dead cell; (III) images of dead cells observed in dark-field with $\lambda_{excitation}$=552nm

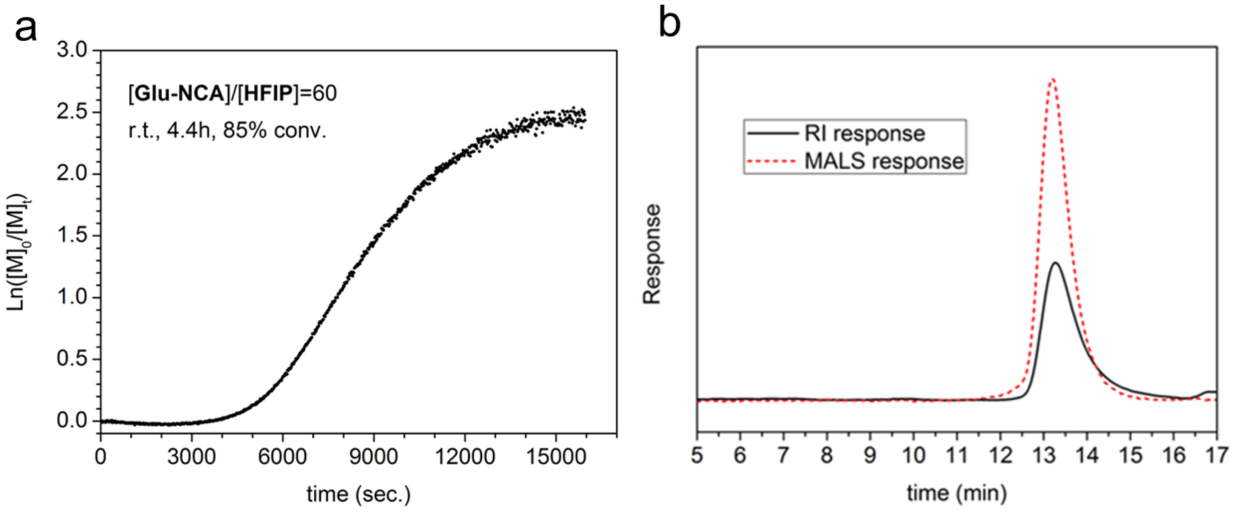

Supplementary Figure 24 Kinetics study of ROP of Glu-NCA in the presence of HFIP. (a) plot of $Ln([M]_0/[M]_t$ vs. time; (b) corresponding SEC traces of obtained polypeptide ($M_{n,SEC}$=3.44×10^4, Đ=1.22); conditions: [Glu-NCA]/[HFIP]=60, $[NCA]_0$=0.19M, 25°C, CH_2Cl_2. Note: A first-order dependence of polymerization rate on the monomer concentration was not observed in kinetics study, indicating a slow initiation of the polymerization (Source data are provided as a Source Data file)

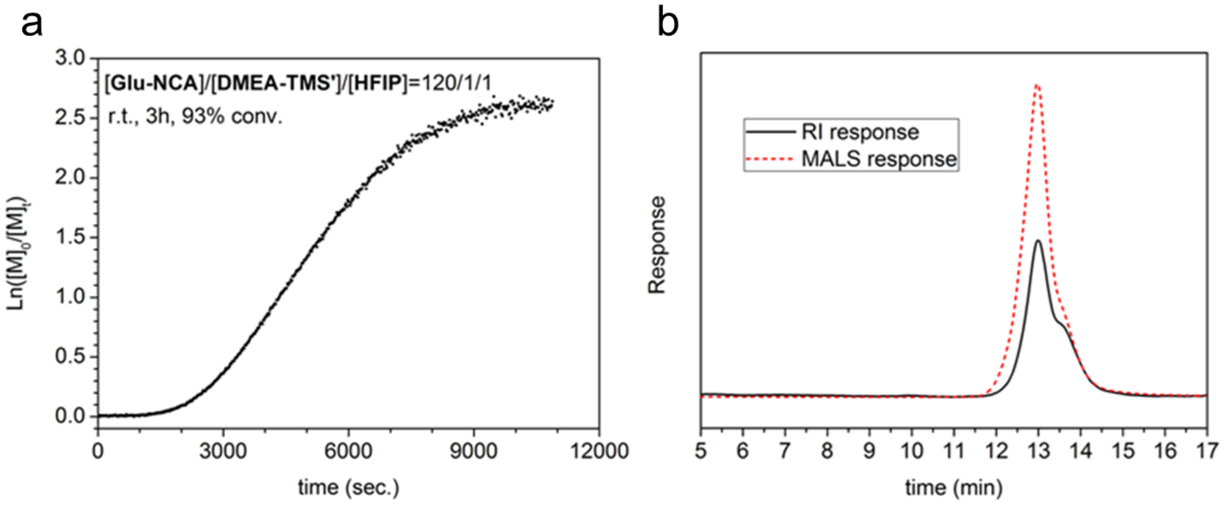

Supplementary Figure 25 Kinetics study of ROP of Glu-NCA initiated by DMES-TMS'/HFIP. (a) plot of $Ln([M]_0/[M]_t$ vs. time; (b) corresponding SEC traces of obtained polypeptide ($M_{n,SEC}$ =2.67×10^5, Đ=1.63); conditions: [Glu-NCA]/[DMES-TMS']/[HFIP] =120/1/1, $[NCA]_0$=0.19M, 25°C, CH_2Cl_2; Note: A first-order dependence of polymerization rate on the monomer concentration was not observed in kinetics study, indicating a slow initiating of the polymerization (Source data are provided as a Source Data file)

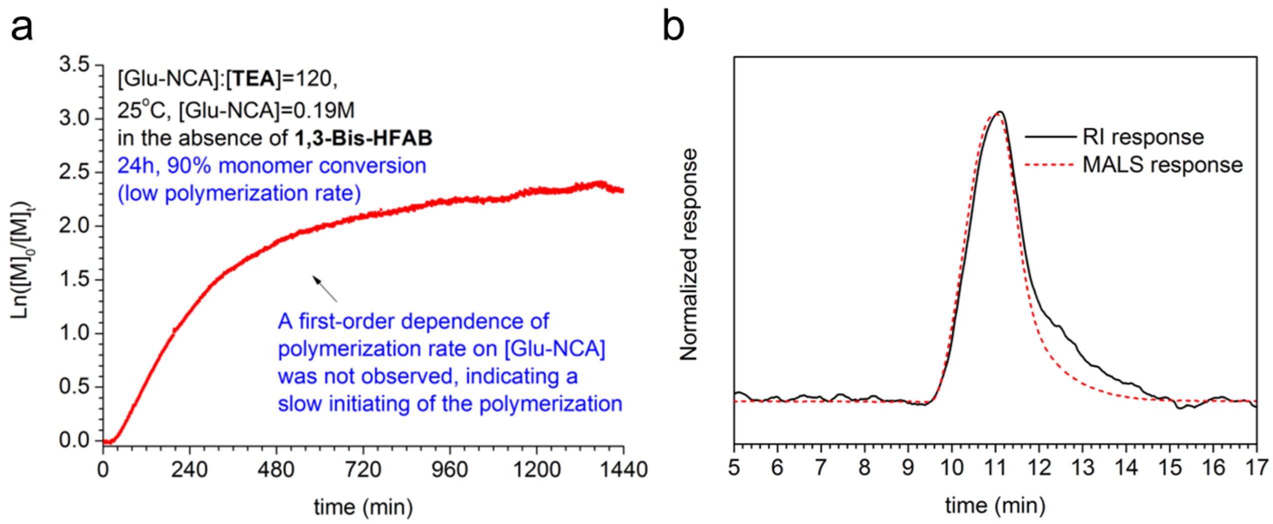

Supplementary Figure 26 Kinetics study of ROP of Glu-NCA initiated by TEA in the absence of 1,3-bis-HFAB. (a) plot of Ln ($[Glu\text{-}NCA]_0/[Glu\text{-}NCA]_t$) vs. time, conditions: [Glu-NCA]/[TEA]=120, $[NCA]_0$=0.19M, 25°C, CH_2Cl_2; (b) corresponding SEC traces of obtained polypeptide ($M_{n,SEC}$=3.92×10^5, Đ=1.80); Note: the $M_{n,SEC}$ is 16 times that of the calculated molecular weight ($M_{n,calcd}$ =2.37×10^4), indicating a slow initiation of the polymerization (Source data are provided as a Source Data file)

Supplementary Table 1 Interaction energies of different complex structures of catalyst/monomer, catalyst/initiator and catalyst/initiator/chain

**Structure Name**	**Ha**	**kcal/mol**	**kJ/mol**
Initiator-1 (Supplementary Figures 27 and 28)	-288.7407	0.00	-
Initiator-2 (lowest energy structure, Supplementary Figures 27 and 28)	-288.7471	-4.02	-16.7871
Monomer (Supplementary Figure 27)	-933.4192	-	-
Catalyst (Supplementary Figure 27)	-1808.4489	-	-
Chain(M)	-1033.6959	-	-
Chain(MM)	-1778.7325	-	-
**Catalyst-Initiator**	-	-	-
Catalyst-Initiator-1(Supplementary Figure 29)	-2097.2821	-58.04	-242.627
Catalyst-Initiator-2(Supplementary Figure 29)	-2097.2756	-53.97	-225.577
Catalyst-Initiator-3 (lowest energy structure, Supplementary Figure 29)	-2097.2832	-58.73	-245.512
**Catalyst-Monomer**	-	-	-
Catalyst-Monomer-1 (lowest energy structure, Supplementary Figure 30)	-2741.9599	-57.61	-240.79
Catalyst-Monomer-2(Supplementary Figure 30)	-2741.9509	-51.96	-217.184
Catalyst-Monomer-3(Supplementary Figure 31)	-2741.9567	-55.60	-232.397
Catalyst-Monomer-4(Supplementary Figure 31)	-2741.9547	-54.34	-227.151
Catalyst-Monomer-5(Supplementary Figure 32)	-2741.9564	-55.41	-231.61
**Catalyst-Initiator-Chain(M)**	-	-	-
Catalyst-Initiator-Chain(M)-1(Supplementary Figure 33)	-2842.2556	-69.53	-290.627
Catalyst-Initiator-Chain(M)-2(Supplementary Figure 33)	-2842.2641	-74.86	-312.923
Catalyst-Initiator-Chain(M)-3(Supplementary Figure 34)	-2842.2665	-76.37	-319.218
Catalyst-Initiator-Chain(M)-4(Supplementary Figure 34)	-2842.2631	-74.23	-310.3
Catalyst-Initiator-Chain(M)-5(Supplementary Figure 35)	-2842.2665	-76.37	-319.218
Catalyst-Initiator-Chain(M)-6(Supplementary Figure 35)	-2842.2606	-72.67	-303.742
Catalyst-Initiator-Chain(M)-7 (lowest energy structure, Supplementary Figure 36)	-2842.2733	-80.63	-337.054
**Catalyst-Initiator-Chain(MM)**	-	-	-
Catalyst-Initiator-Chain(MM)-1 (Supplementary Figure 37)	-3587.2639	-51.77	-216.397

Catalyst-Initiator-Chain(MM)-2 (Supplementary Figure 38)	-3587.2603	-49.51	-206.954
Catalyst-Initiator-Chain(MM)-3 (Supplementary Figure 39)	-3587.2251	-27.42	-114.625
Catalyst-Initiator-Chain(MM)-4 (Supplementary Figure 40)	-3587.2599	-49.26	-205.905
Catalyst-Initiator-Chain(MM)-5 (Supplementary Figure 41)	-3587.2609	-49.89	-208.528
Catalyst-Initiator-Chain(MM)-6 (lowest energy structure, Supplementary Figure 42)	-3587.2769	-59.93	-250.495
Catalyst-Initiator-Chain(MM)-7 (Supplementary Figure 43)	-3587.2624	-50.83	-212.462
Catalyst-Initiator-Chain(MM)-8 (Supplementary Figure 44)	-3587.2592	-48.82	-204.069
Catalyst-Initiator-Chain(MM)-9 (Supplementary Figure 45)	-3587.2617	-50.39	-210.626
Catalyst-Initiator-Chain(MM)-10 (Supplementary Figure 46)	-3587.2624	-50.83	-212.462
Catalyst-Initiator-Chain(MM)-11 (Supplementary Figure 47)	-3587.2711	-56.29	-235.282
Catalyst-Initiator-Chain(MM)-12 (Supplementary Figure 48)	-3587.2645	-52.15	-217.97
Catalyst-Initiator-Chain(MM)-13 (Supplementary Figure 49)	-3587.2600	-49.32	-206.167
Catalyst-Initiator-Chain(MM)-14 (Supplementary Figure 50)	-3587.2678	-54.22	-226.626

Note: The data in supplementary table 1 are obtained from the computational study in which L-Glu-NCA is used. All the configurations of complexes of catalyst/monomer, catalyst/initiator and catalyst/initiator/chain are provided (see Supplementary Figures 27-50). Beside L-Glu-NCA, the D-Glu-NCA was also used for the computational study and the corresponding configurations of complexes are provided in Supplementary Figures 27 and 30-50 for reference.

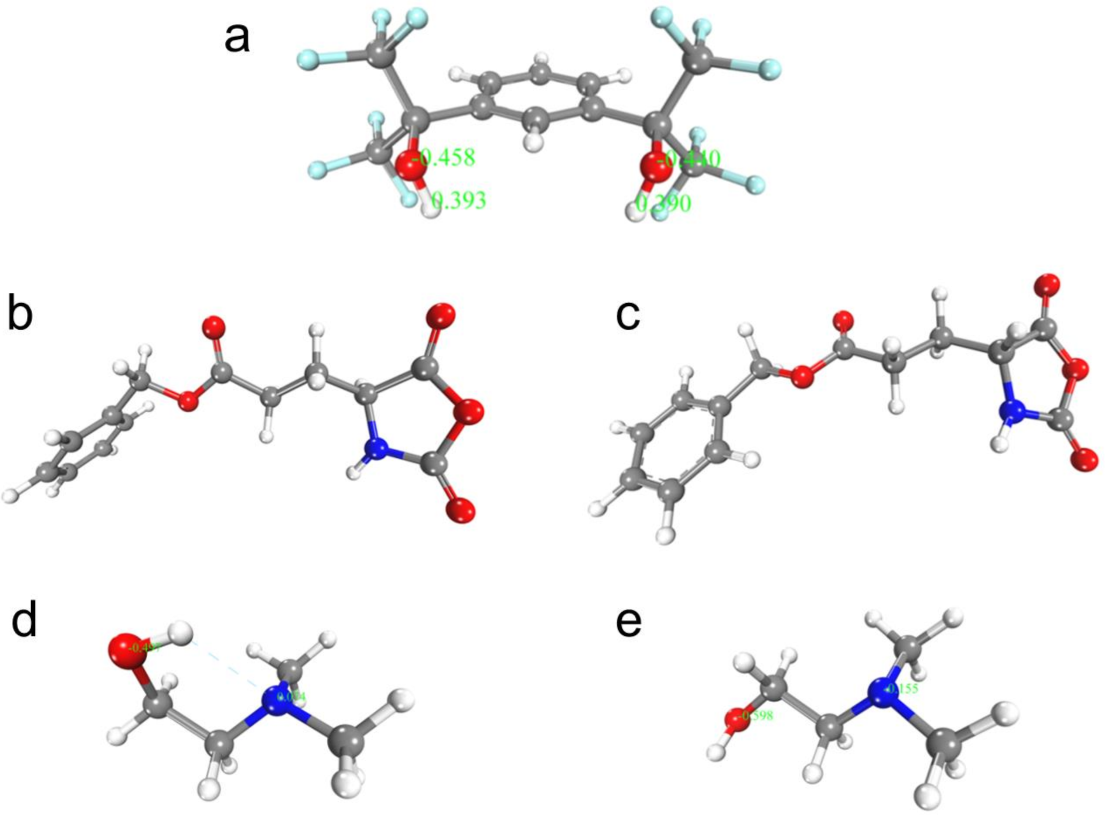

Supplementary Figure 27 The configurations of monomer, catalyst and initiator in Supplementary Table 1. **a** catalyst; **b** L-Glu-NCA monomer; **c** D-Glu-NCA monomer; **d** low-energy configuration of initiator; **e** high-energy configuration of initiator

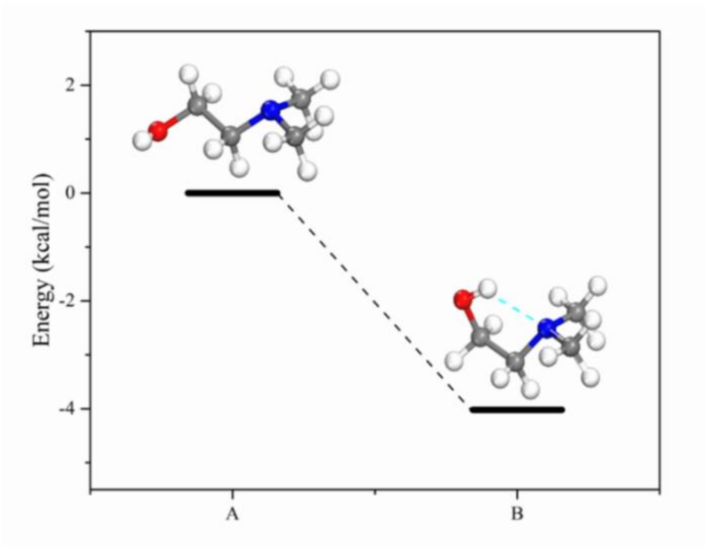

Supplementary Figure 28 Relative energies of two configurations of DMEA molecule according to DFT calculation (ΔE=-4.02 kcal/mol)

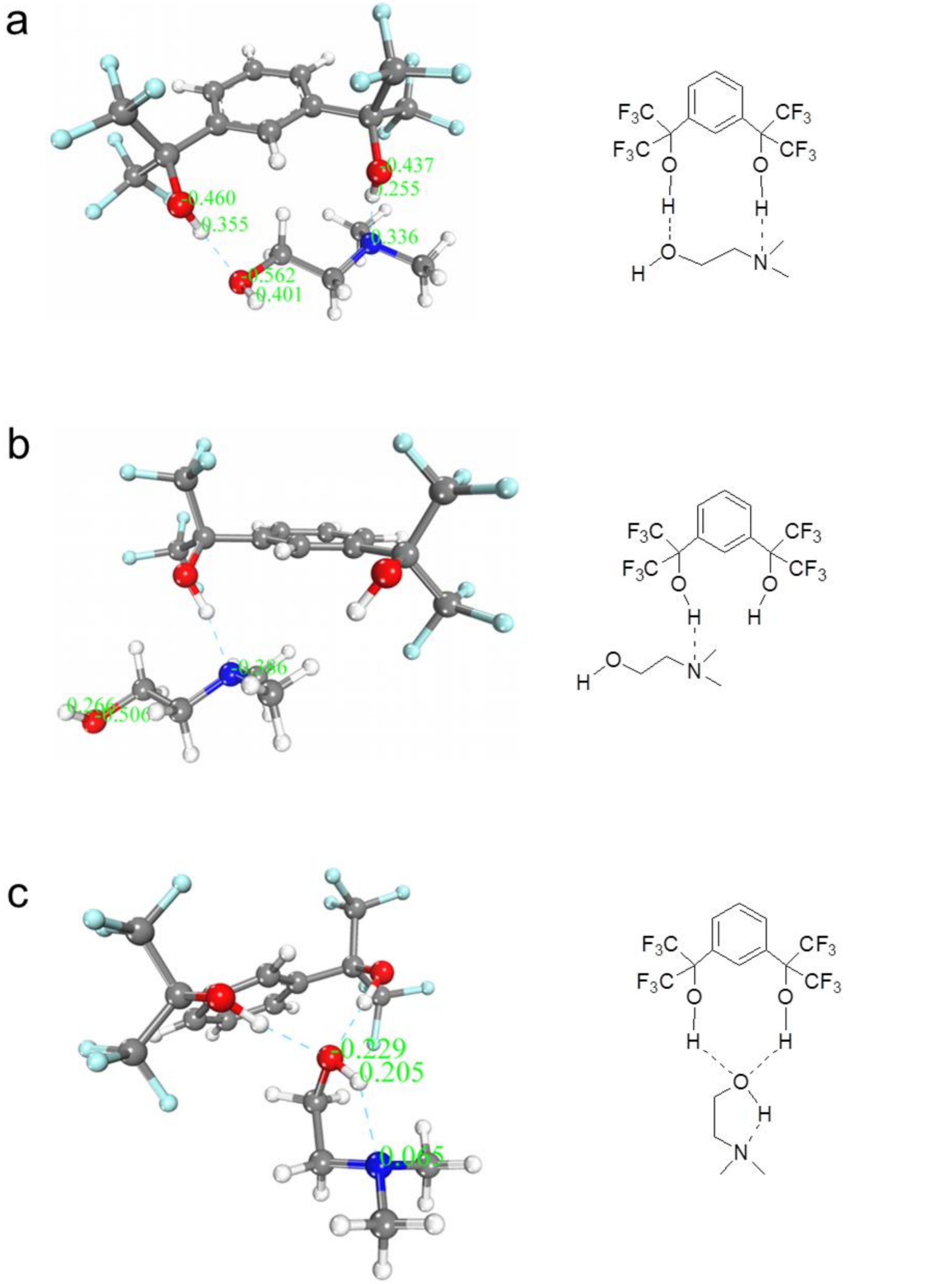

Supplementary Figure 29 The configurations of catalyst-initiator complexes in Supplementary Table 1. **a** catalyst-initiator-1; **b** catalyst-initiator-2; **c** catalyst-initiator-3

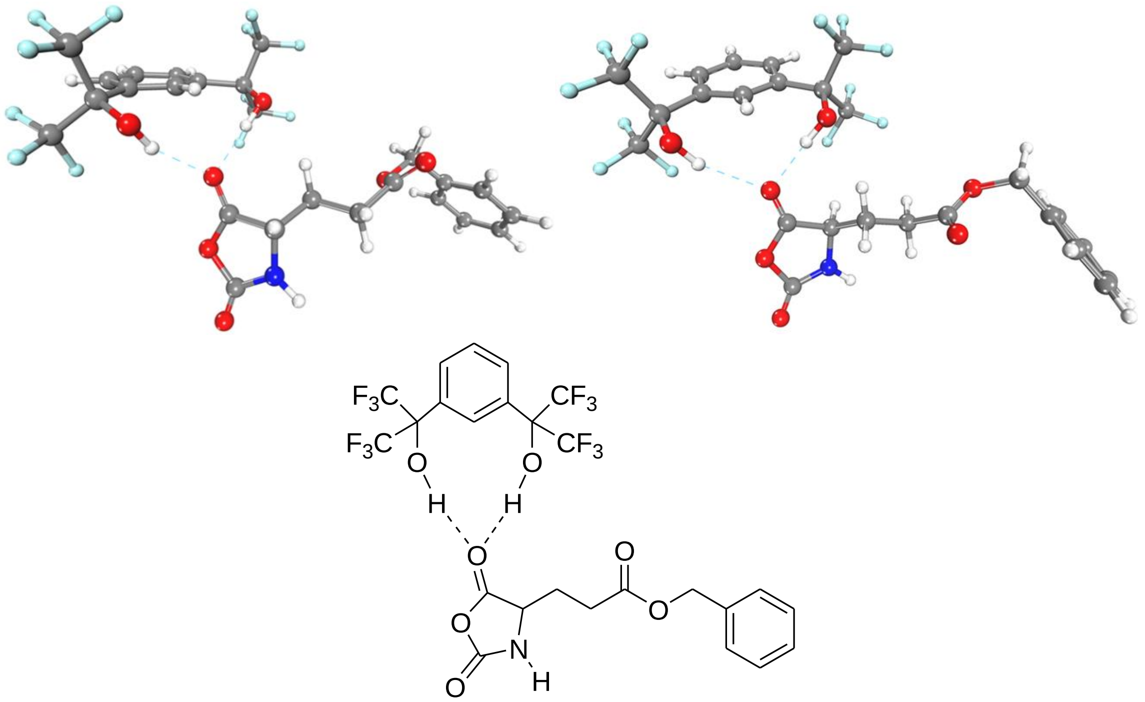

Catalyst-Monomer-1

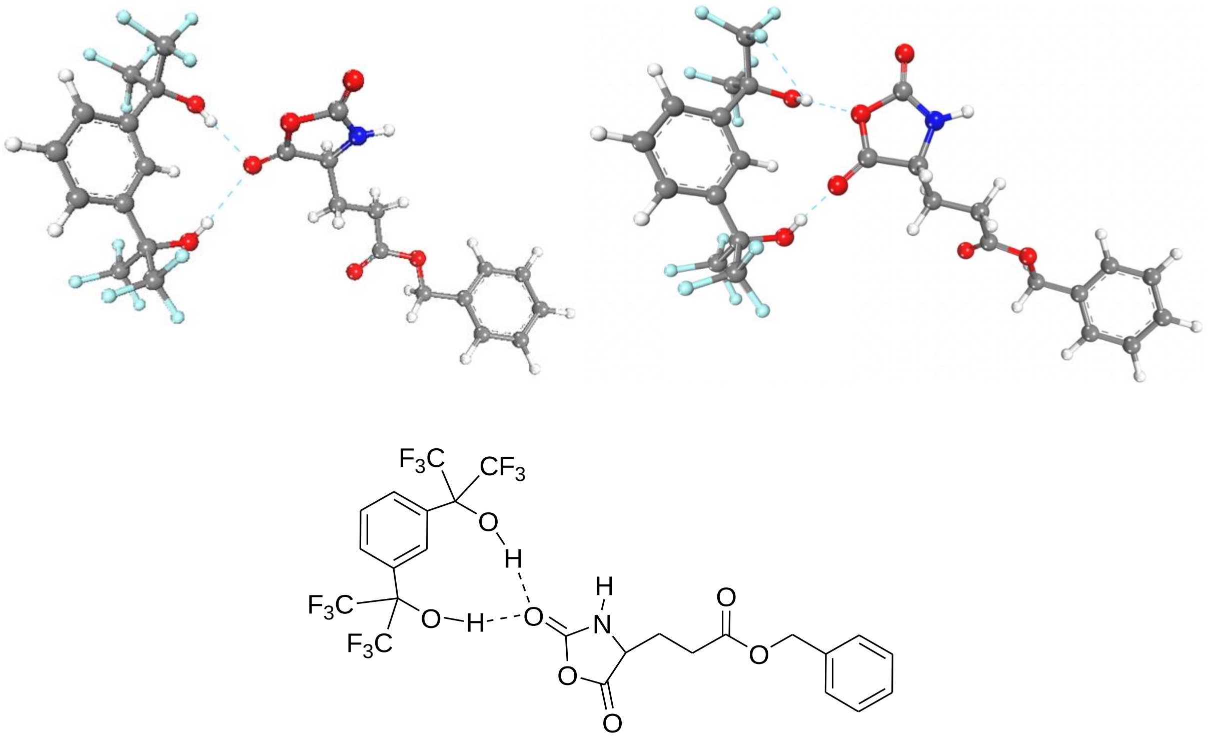

Catalyst-Monomer-2

Supplementary Figure 30 The configurations of catalyst-monomer-1 and catalyst-monomer-2 complexes in Supplementary Table 1 (on the left is the configurations of complexes involving D-Glu-NCA)

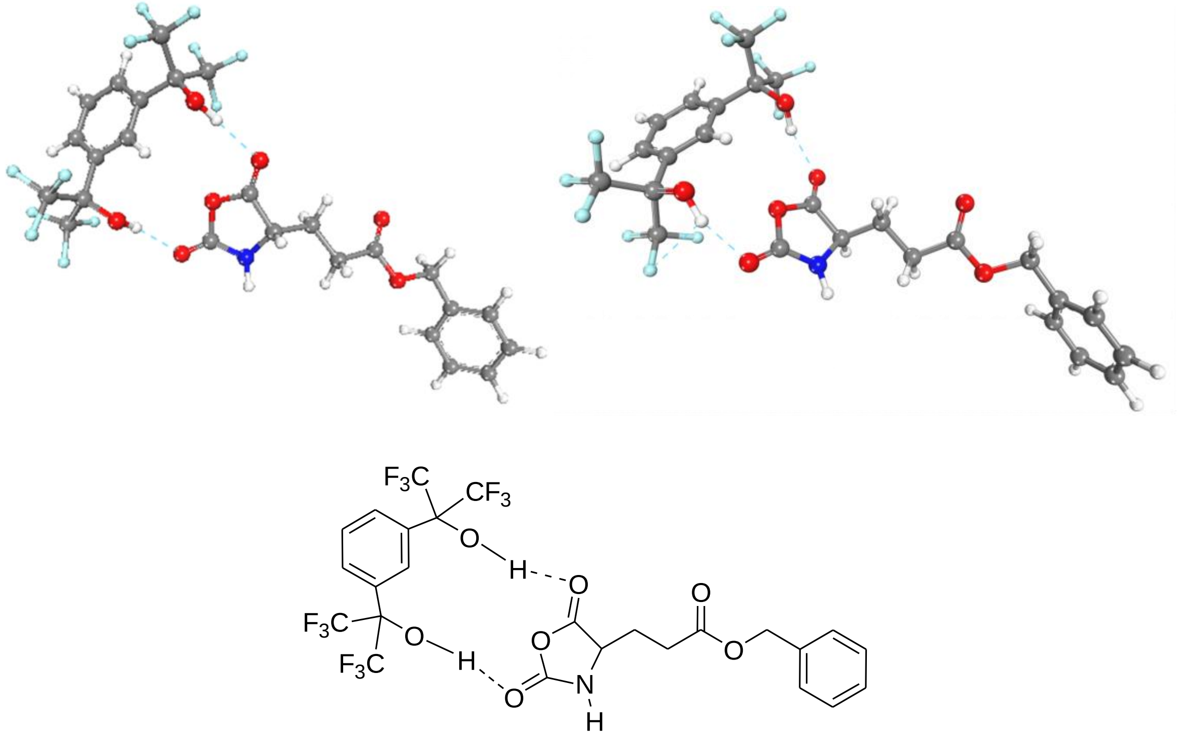

Catalyst-Monomer-3

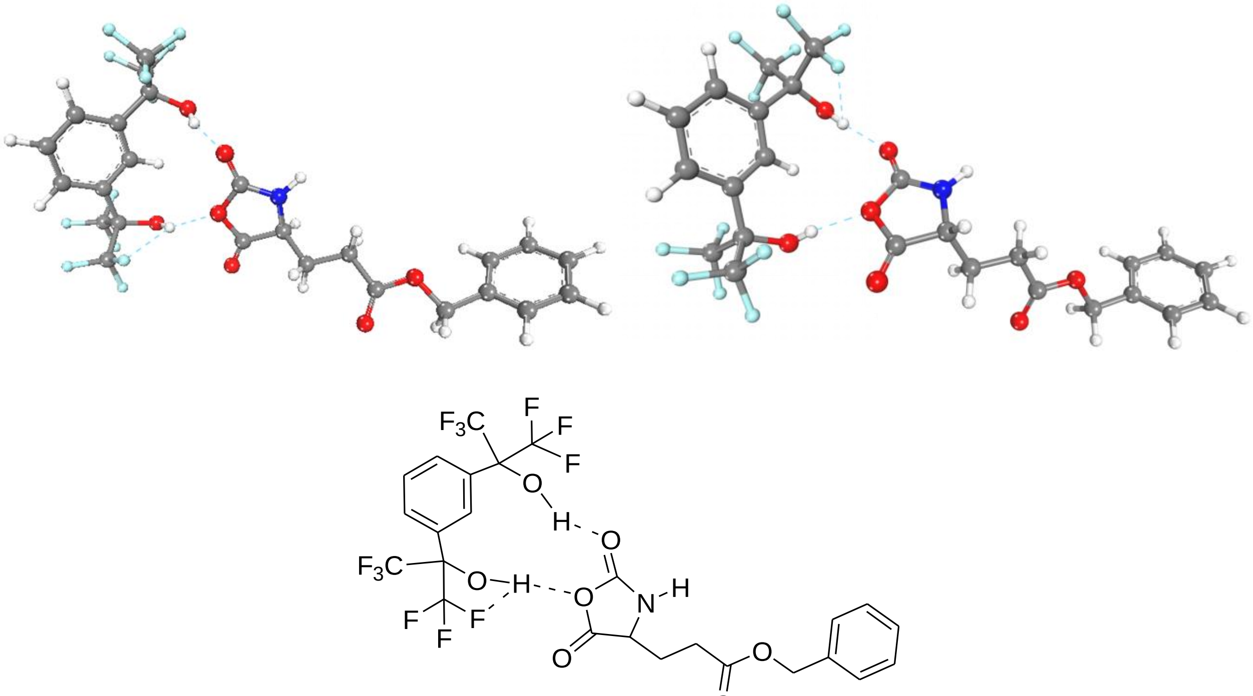

Catalyst-Monomer-4

Supplementary Figure 31 The configurations of catalyst-monomer-3 and catalyst-monomer-4 complexes in Supplementary Table 1 (on the left is the configurations of complexes involving D-Glu-NCA)

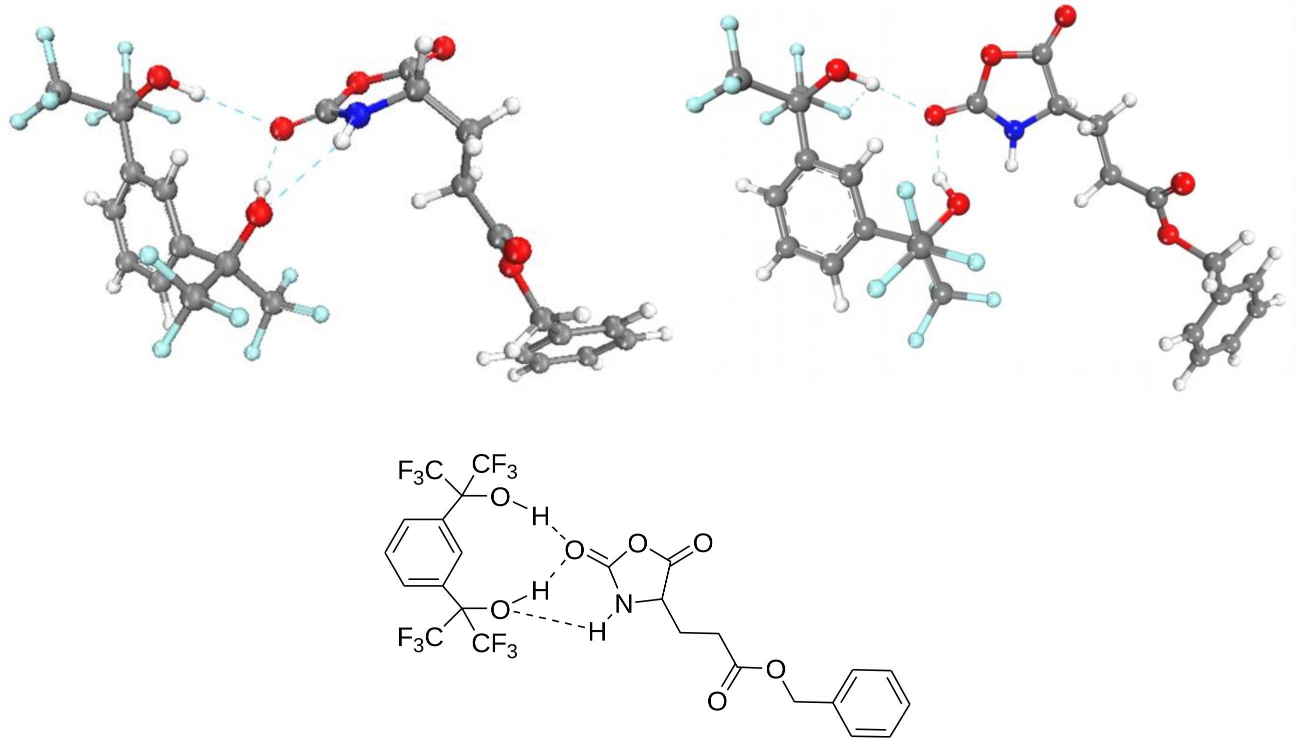

Catalyst-Monomer-5

Supplementary Figure 32 The configuration of catalyst-monomer-5 complex in Supplementary Table 1 (on the left is the configuration of complex involving D-Glu-NCA)

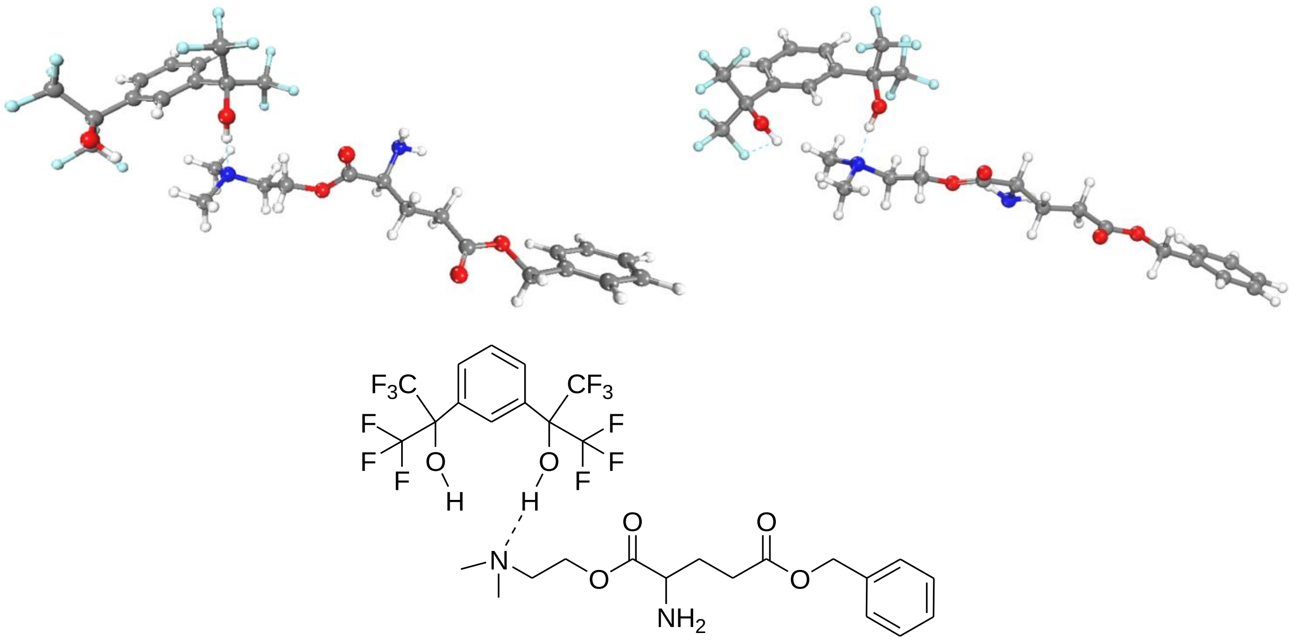

Catalyst-Initiator-Chain(M)-1

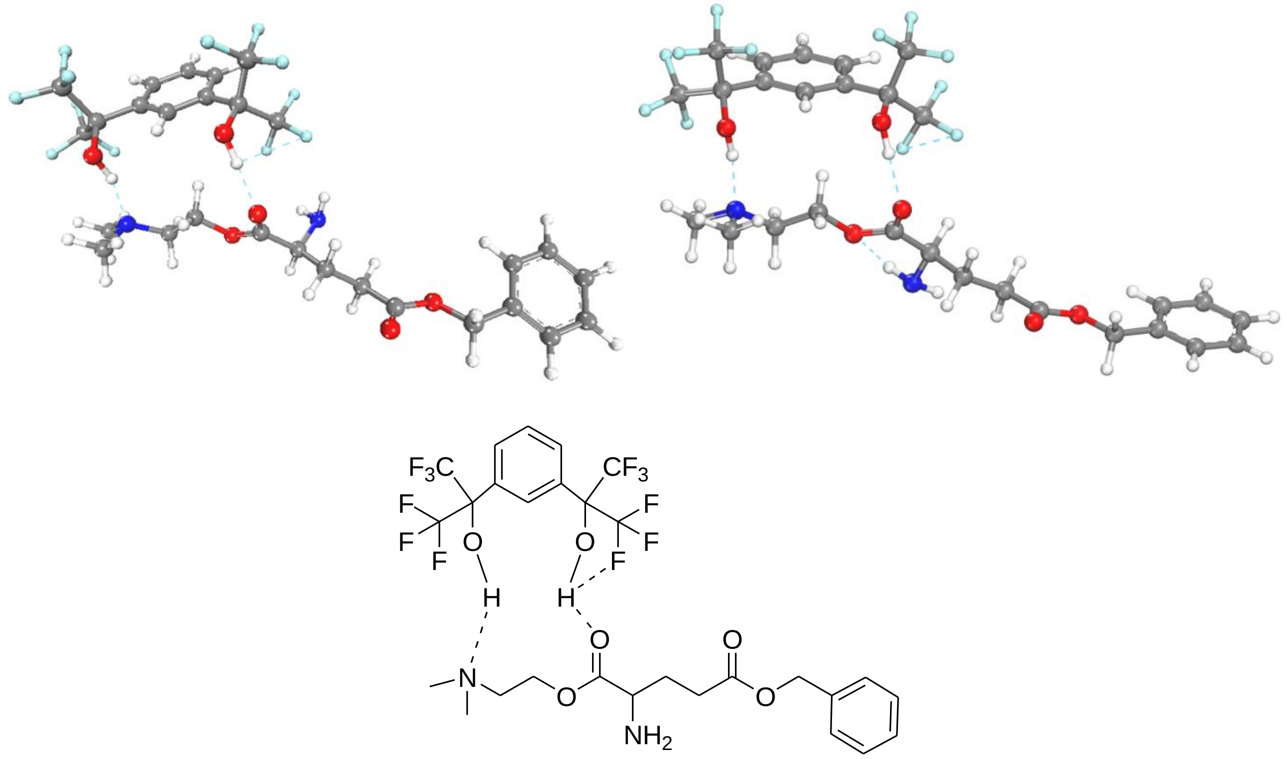

Catalyst-Initiator-Chain(M)-2

Supplementary Figure 33 The configurations of catalyst-initiator-chain(M)-1 and catalyst-initiator-chain(M)-2 complexes in Supplementary Table 1 (chain(M) represents one inserted monomer; on the left is the configurations of complexes involving D-Glu-NCA)

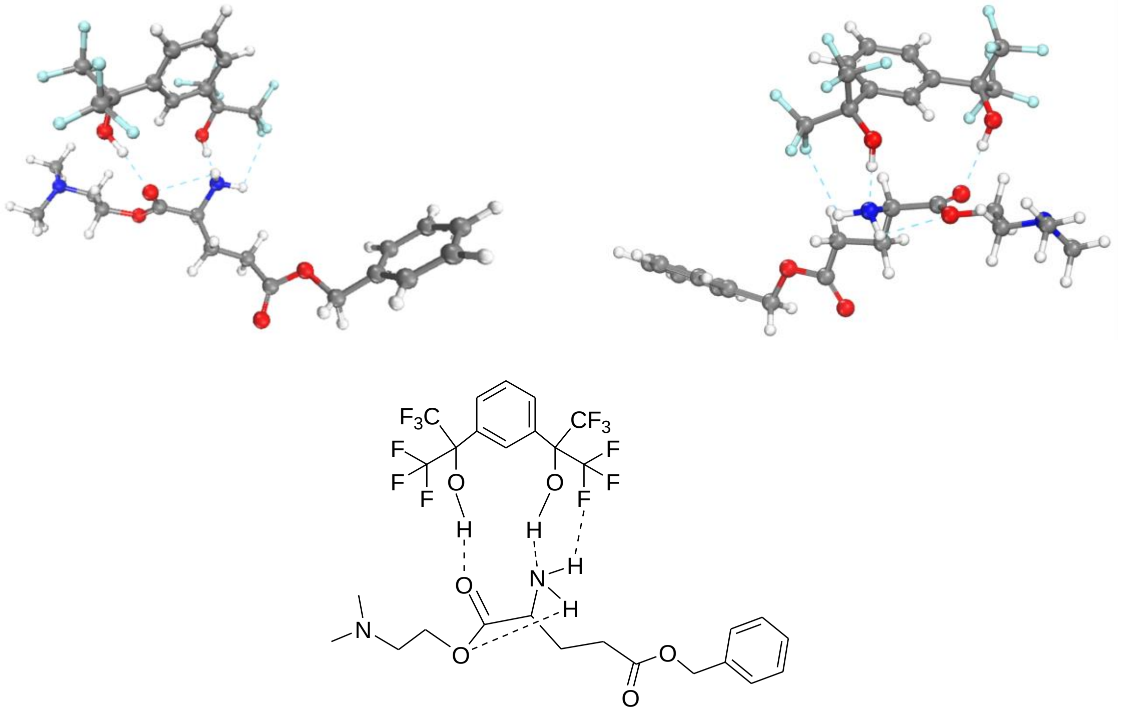

Catalyst-Initiator-Chain(M)-3

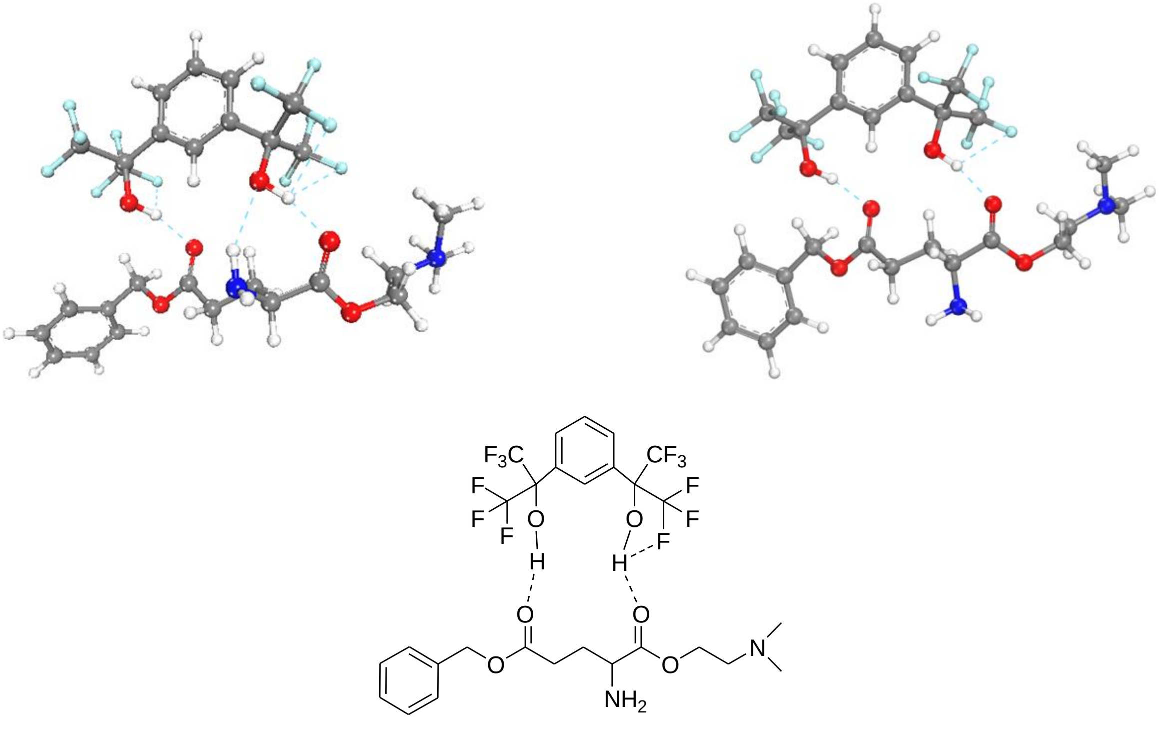

Catalyst-Initiator-Chain(M)-4

Supplementary Figure 34 The configurations of catalyst-initiator-chain(M)-3 and catalyst-initiator-chain(M)-4 complexes in Supplementary Table 1 (chain(M) represents one inserted monomer; on the left is the configurations of complexes involving D-Glu-NCA)

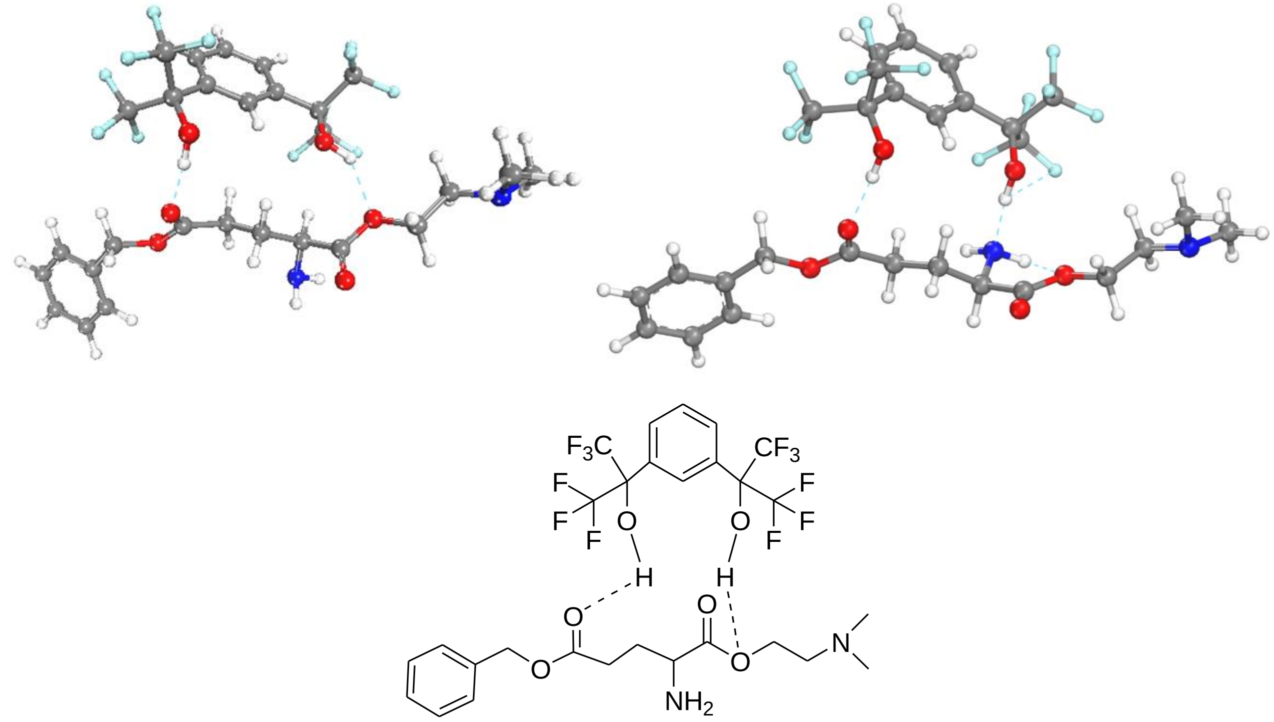

Catalyst-Initiator-Chain(M)-5

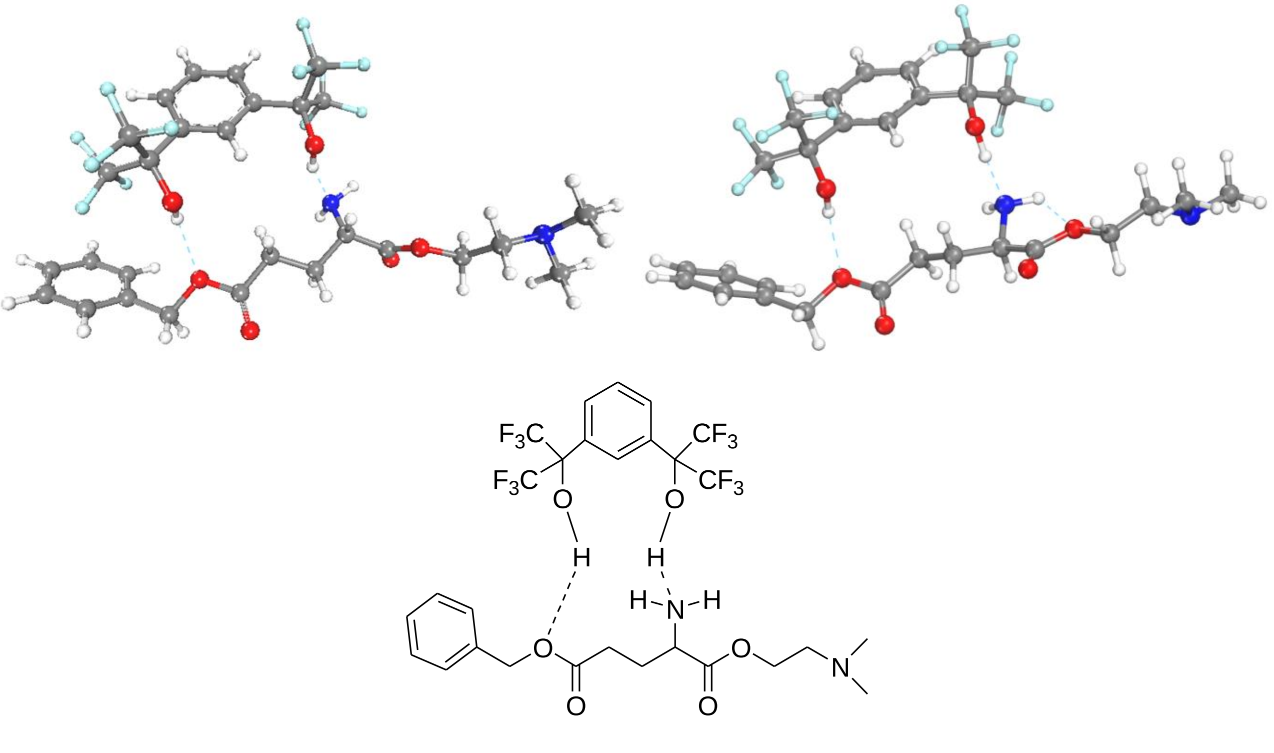

Catalyst-Initiator-Chain(M)-6

Supplementary Figure 35 The configurations of catalyst-initiator-chain(M)-5 and catalyst-initiator-chain(M)-6 complexes in Supplementary Table 1 (chain(M) represents one inserted monomer; on the left is the configurations of complexes involving D-Glu-NCA)

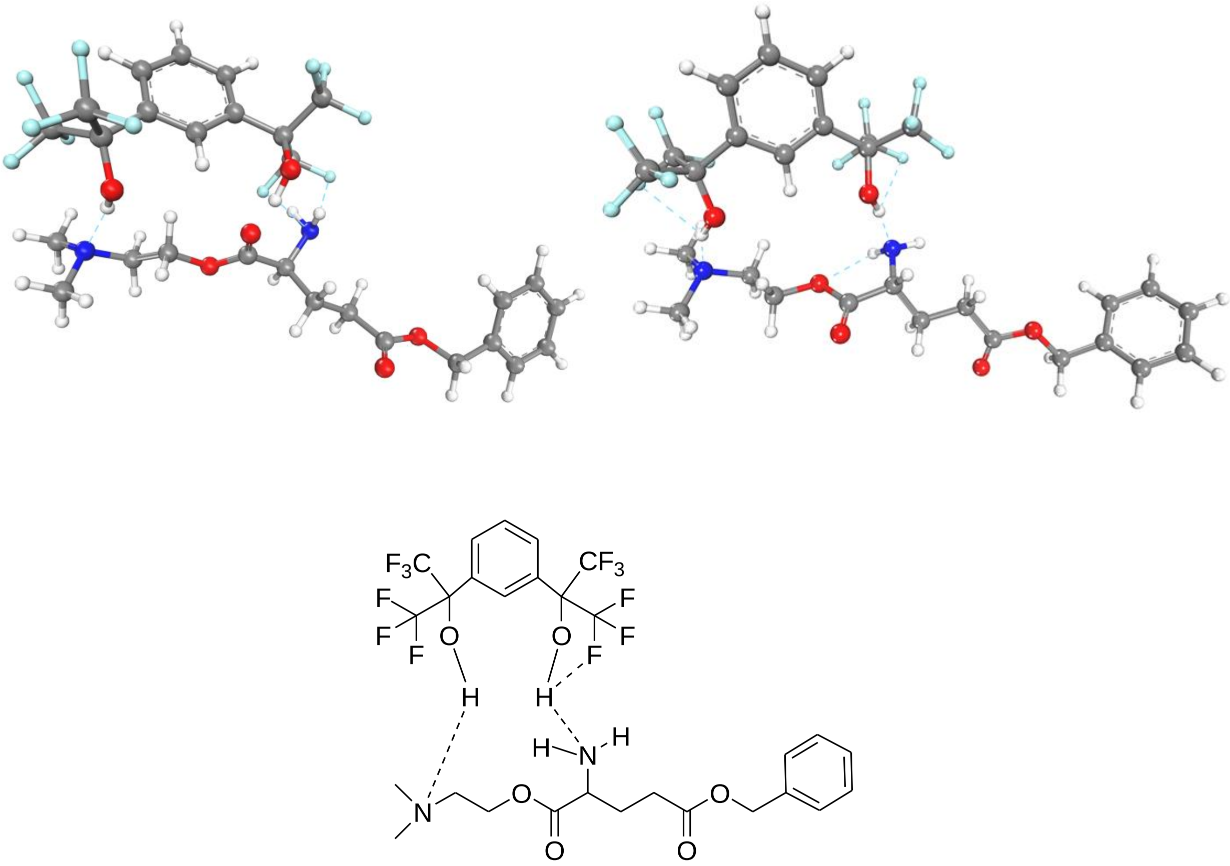

Catalyst-Initiator-Chain(M)-7

Supplementary Figure 36 The configuration of catalyst-initiator-chain(M)-7 complex in Supplementary Table 1 (chain(M) represents one inserted monomer; on the left is the configuration of complex involving D-Glu-NCA)

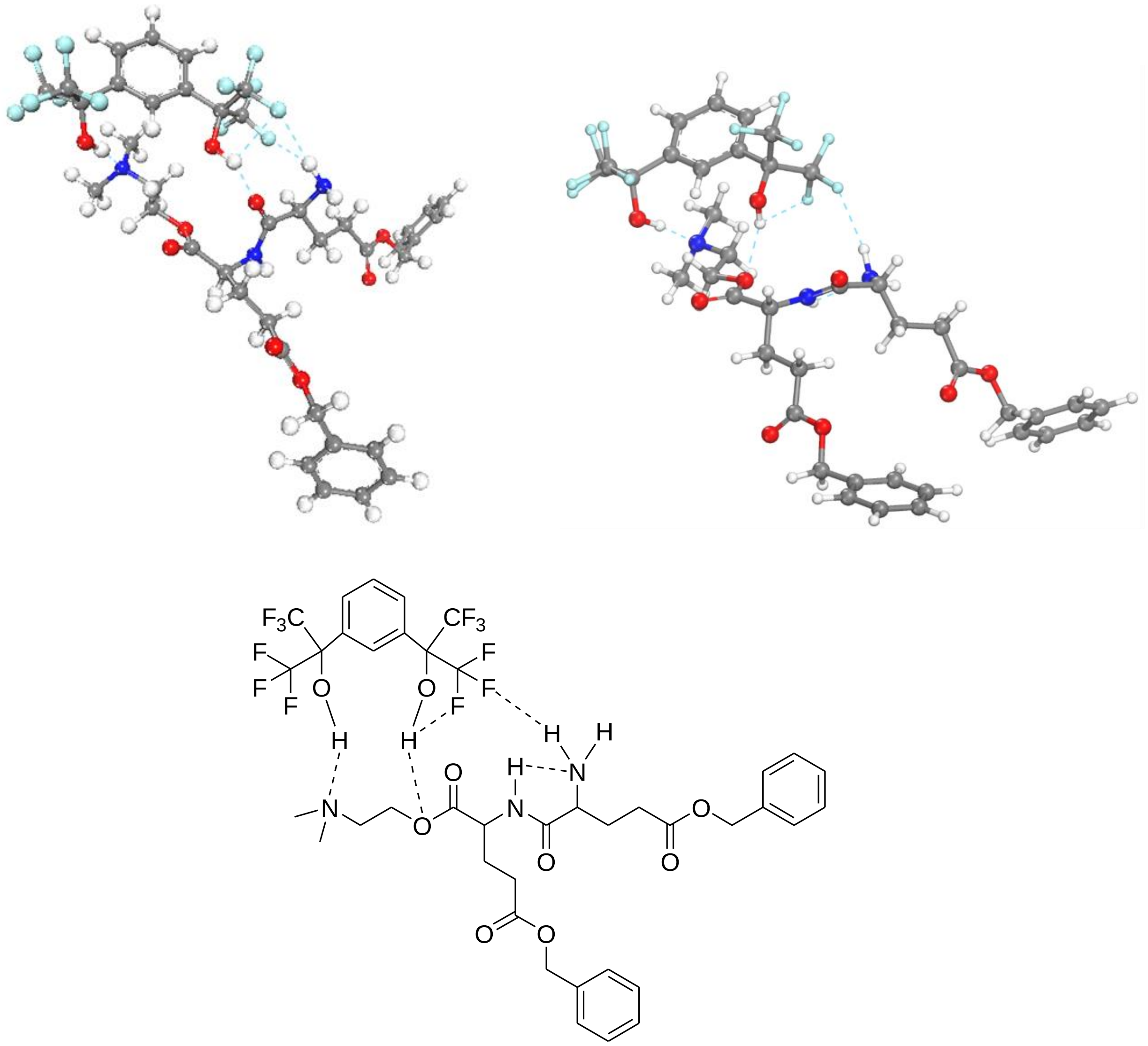

Catalyst-Initiator-Chain(MM)-1

Supplementary Figure 37 The configuration of catalyst-initiator-chain(MM)-1 complex in Supplementary Table 1 (chain(MM) represents two inserted monomer; on the left is the configuration of complex involving D-Glu-NCA)

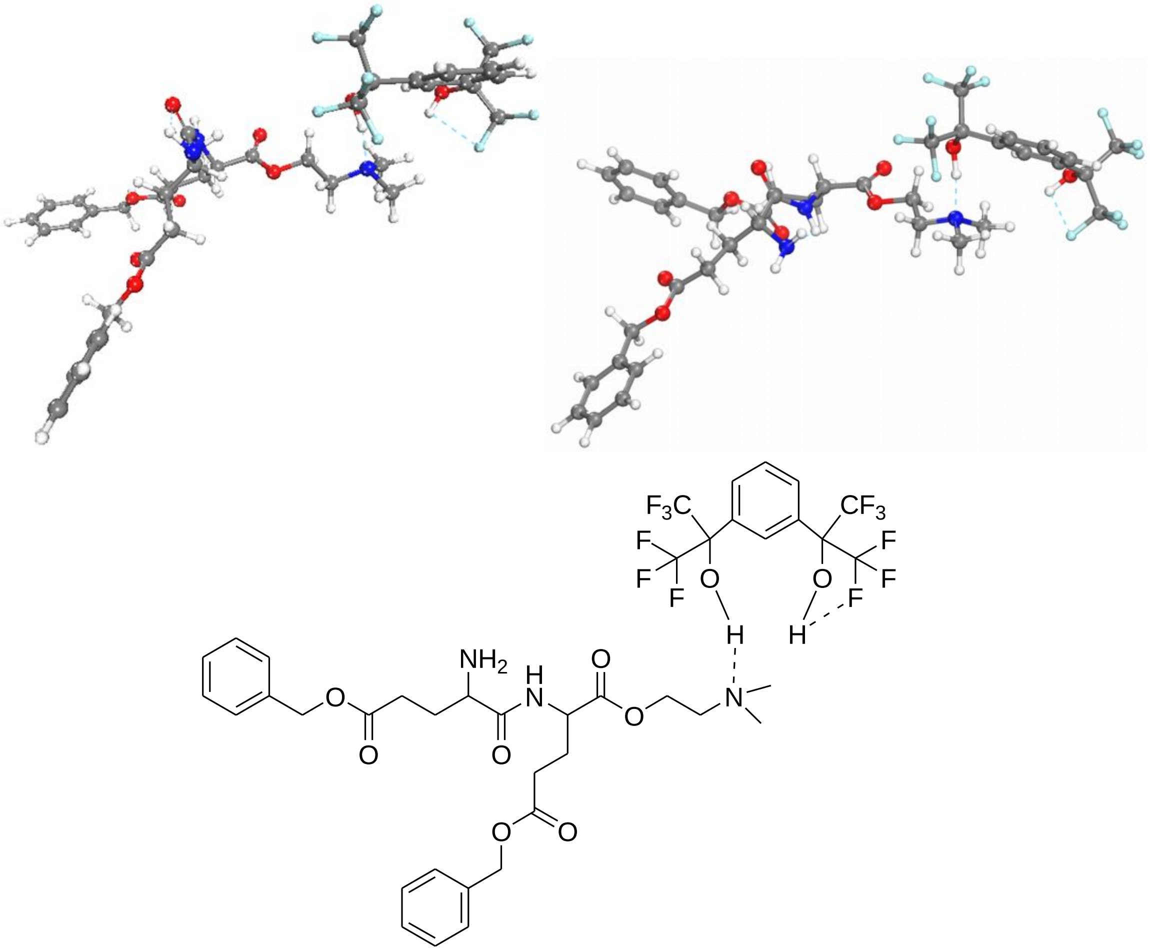

Catalyst-Initiator-Chain(MM)-2

Supplementary Figure 38 The configuration of catalyst-initiator-chain(MM)-2 complex in Supplementary Table 1 (chain(MM) represents two inserted monomer; on the left is the configuration of complex involving D-Glu-NCA)

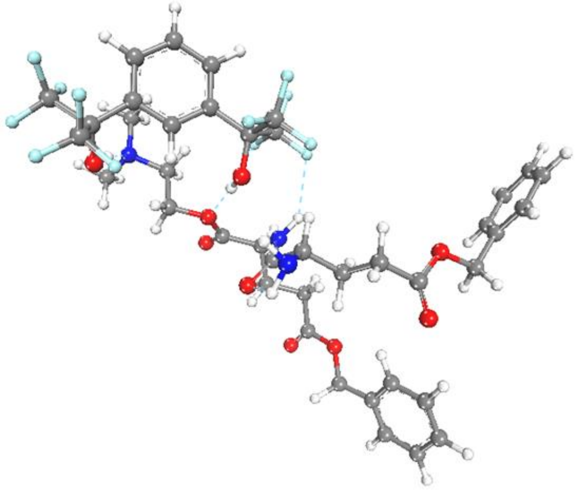

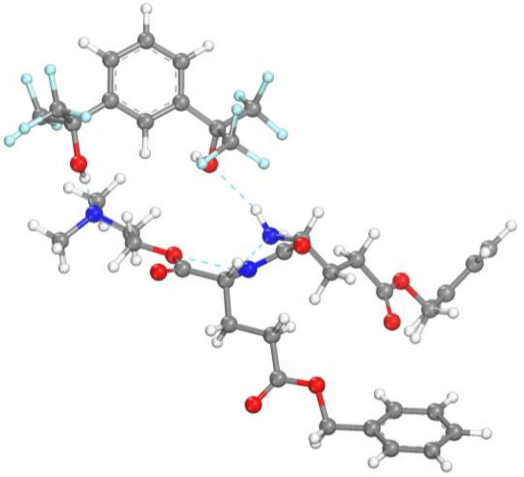

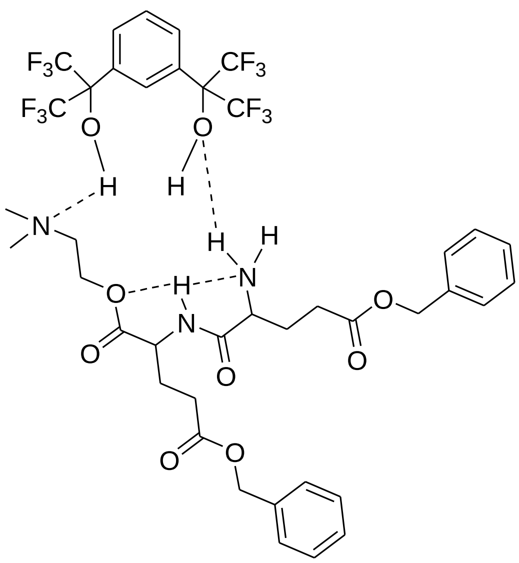

Catalyst-Initiator-Chain(MM)-3

Supplementary Figure 39 The configuration of catalyst-initiator-chain(MM)-3 complex in Supplementary Table 1 (chain(MM) represents two inserted monomer; on the left is the configuration of complex involving D-Glu-NCA)

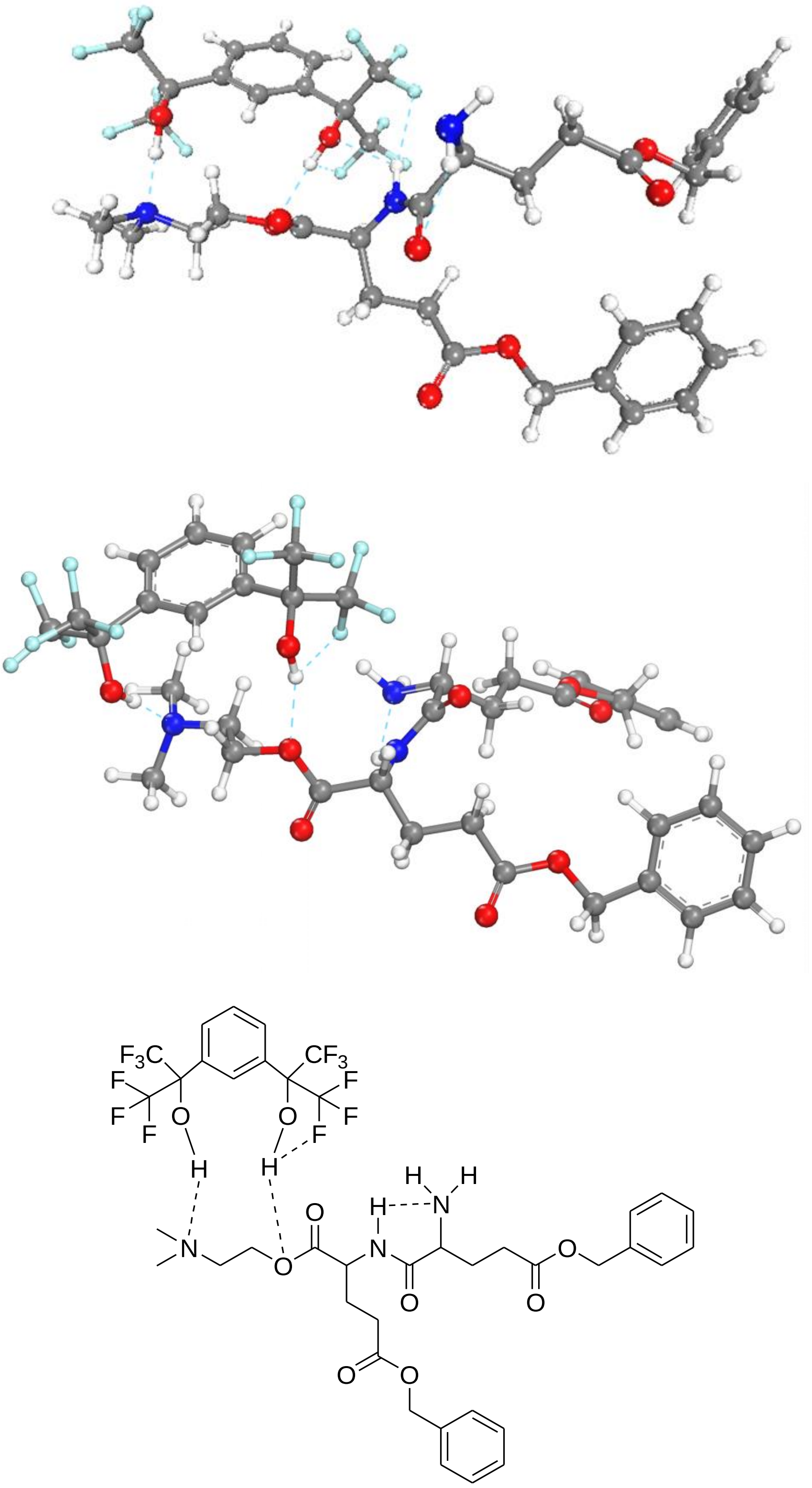

Catalyst-Initiator-Chain(MM)-4

Supplementary Figure 40 The configuration of catalyst-initiator-chain(MM)-4 complex in Supplementary Table 1 (chain(MM) represents two inserted monomer; below is the configuration of complex involving D-Glu-NCA)

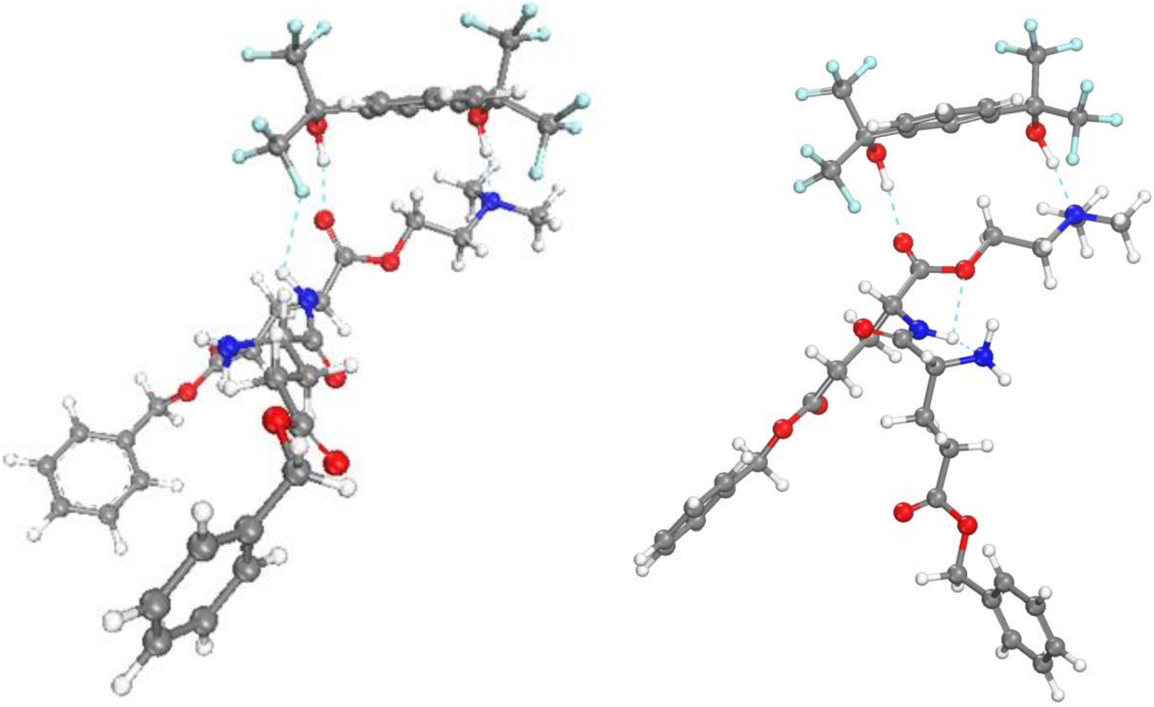

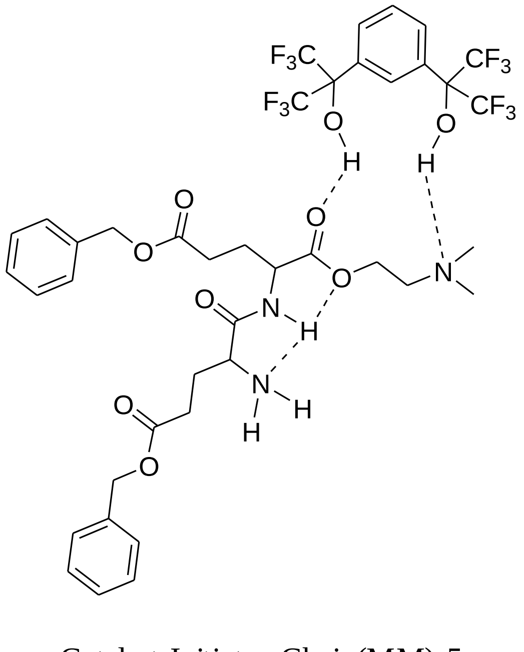

Catalyst-Initiator-Chain(MM)-5

Supplementary Figure 41 The configuration of catalyst-initiator-chain(MM)-5 complex in Supplementary Table 1 (chain(MM) represents two inserted monomer; on the left is the configuration of complex involving D-Glu-NCA)

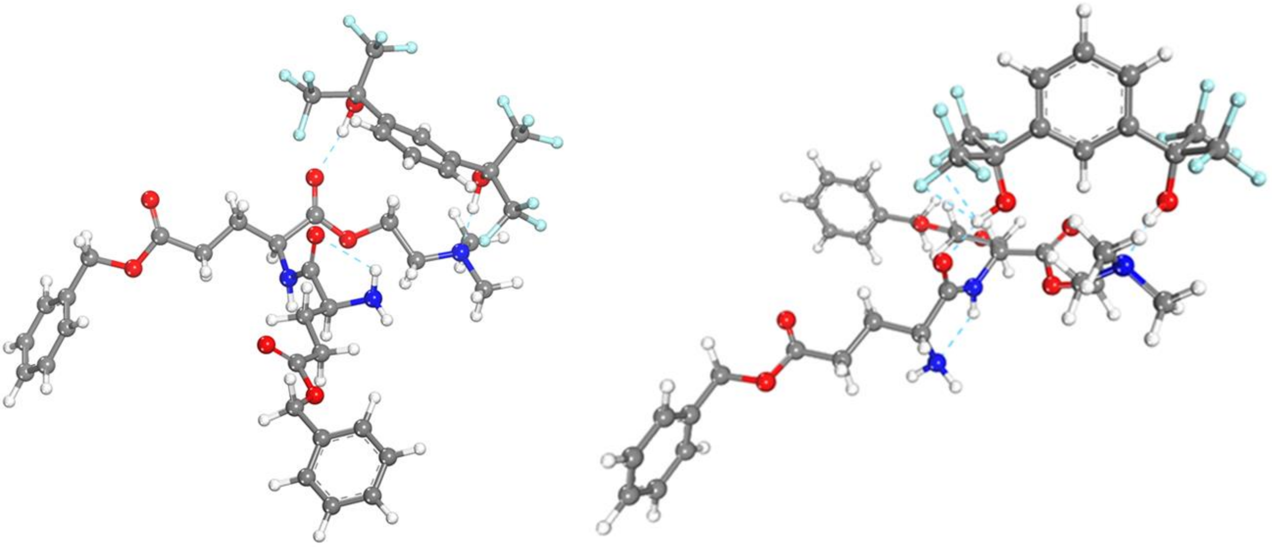

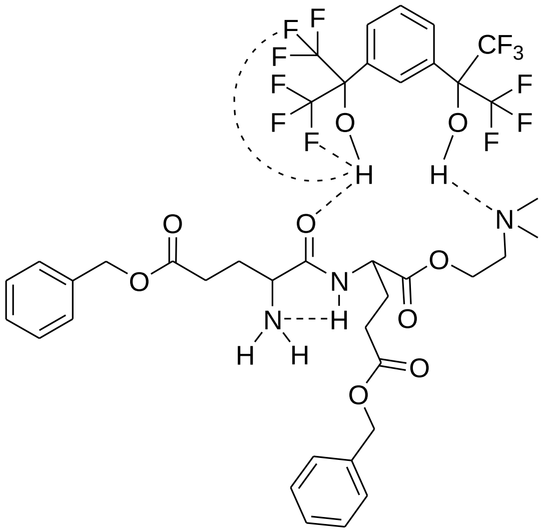

Catalyst-Initiator-Chain(MM)-6

Supplementary Figure 42 The configuration of catalyst-initiator-chain(MM)-6 complex in Supplementary Table 1 (chain(MM) represents two inserted monomer; on the left is the configuration of complex involving D-Glu-NCA)

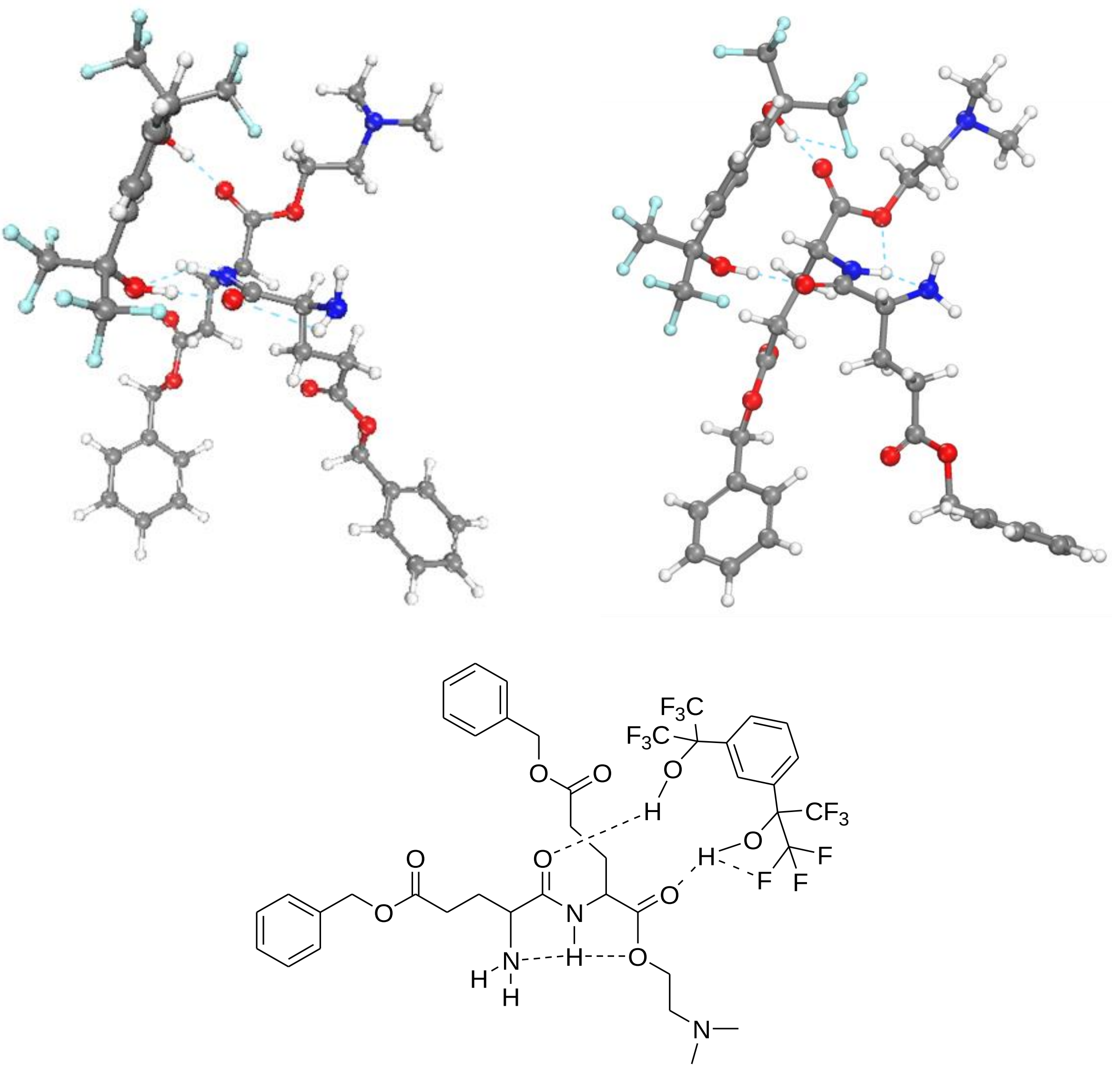

Catalyst-Initiator-Chain(MM)-7

Supplementary Figure 43 The configuration of catalyst-initiator-chain(MM)-7 complex in Supplementary Table 1 (chain(MM) represents two inserted monomer; on the left is the configuration of complex involving D-Glu-NCA)

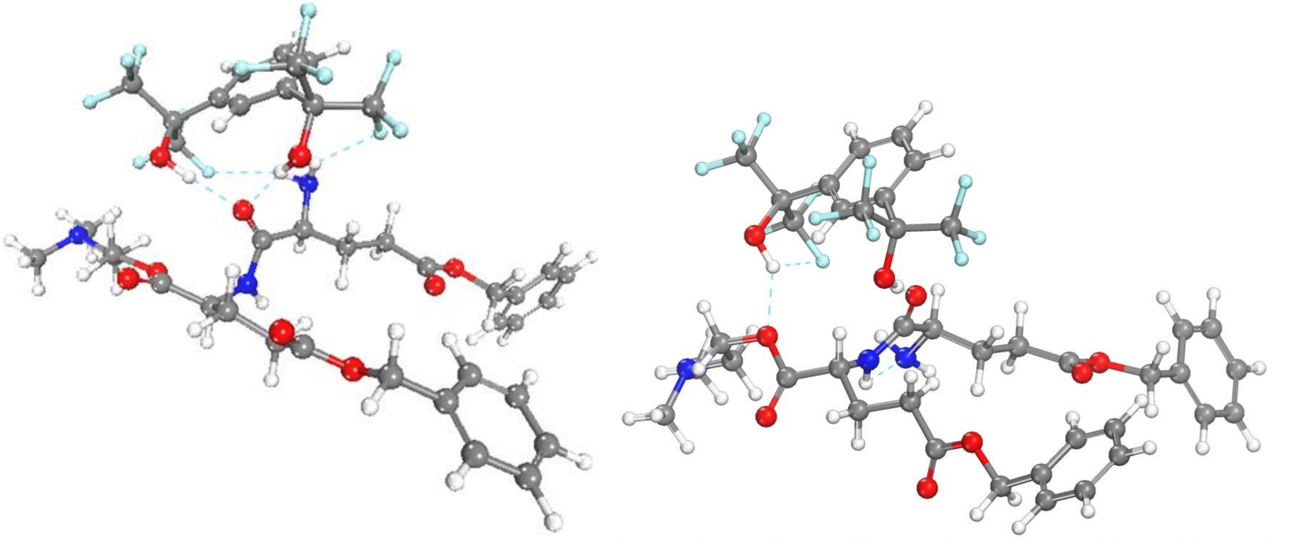

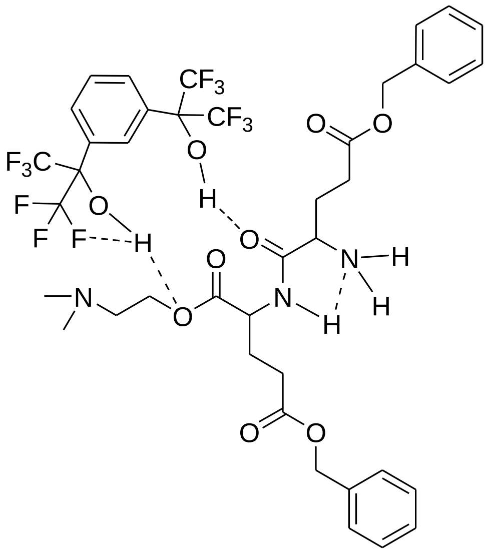

Catalyst-Initiator-Chain(MM)-8

Supplementary Figure 44 The configuration of catalyst-initiator-chain(MM)-8 complex in Supplementary Table 1 (chain(MM) represents two inserted monomer; on the left is the configuration of complex involving D-Glu-NCA)

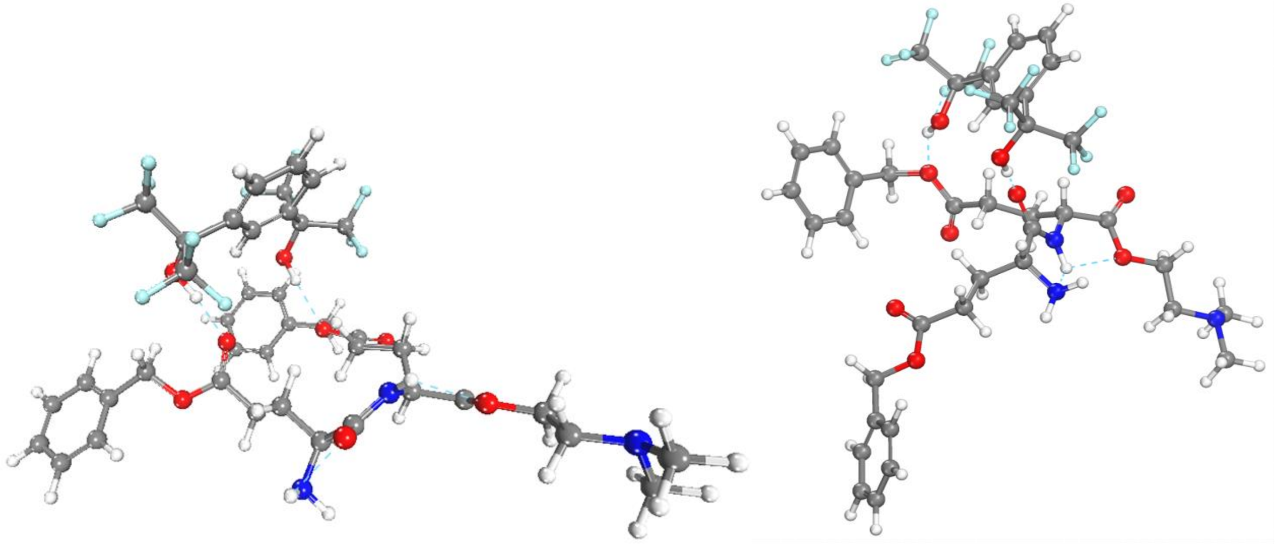

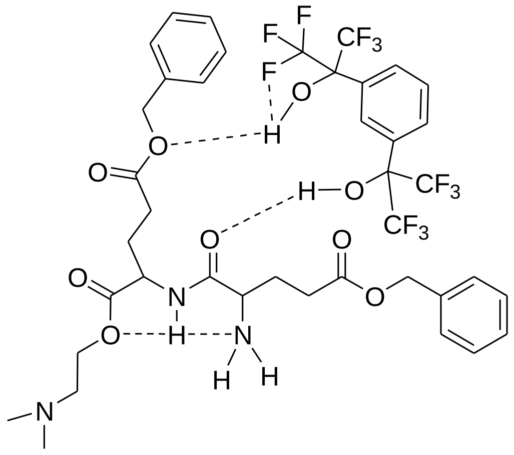

Catalyst-Initiator-Chain(MM)-9

Supplementary Figure 45 The configuration of catalyst-initiator-chain(MM)-9 complex in Supplementary Table 1 (chain(MM) represents two inserted monomer, on the left is the configuration of complex involving D-Glu-NCA)

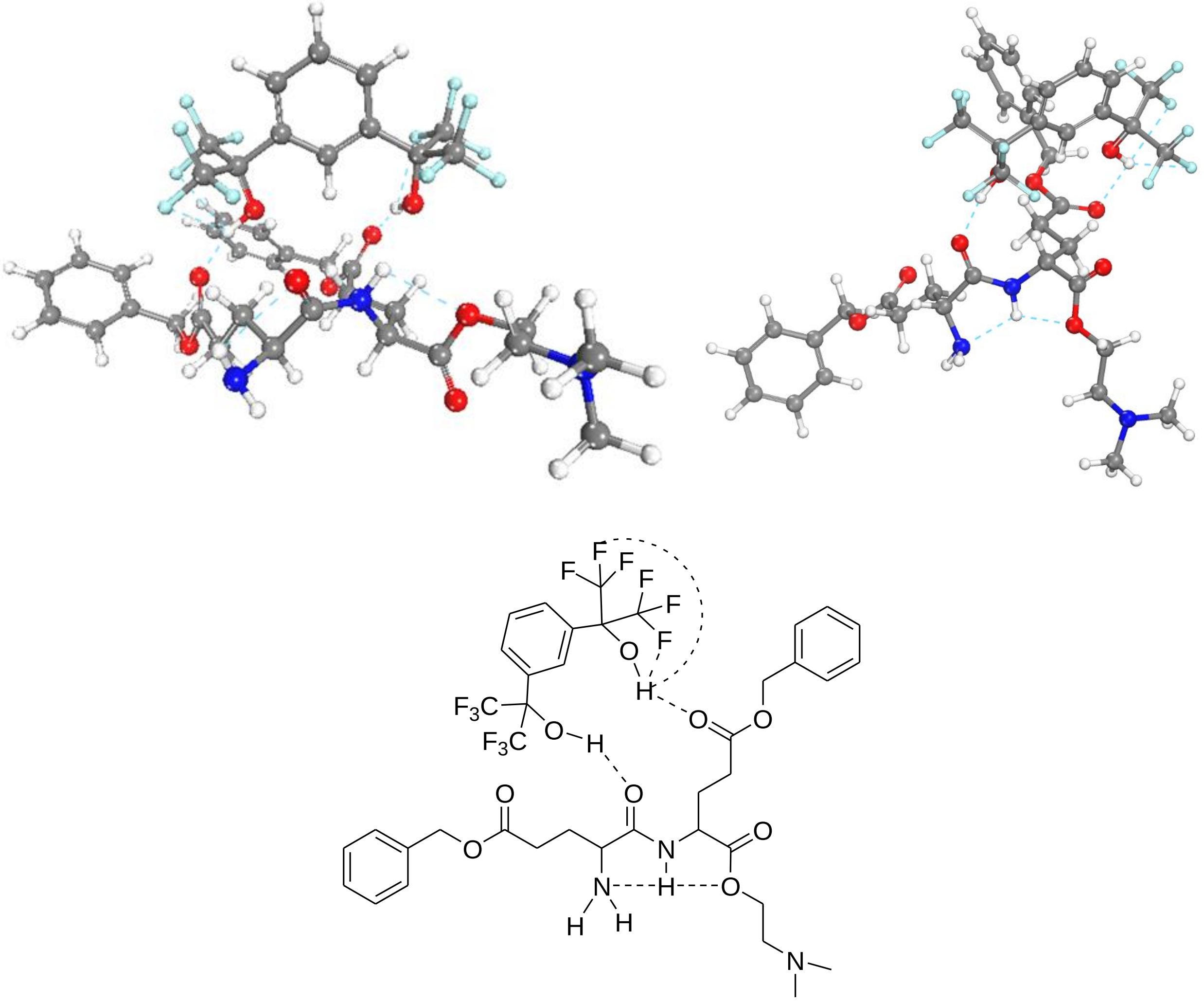

Catalyst-Initiator-Chain(MM)-10

Supplementary Figure 46 The configuration of catalyst-initiator-chain(MM)-10 complex in Supplementary Table 1 (chain(MM) represents two inserted monomer; on the left is the configuration of complex involving D-Glu-NCA)

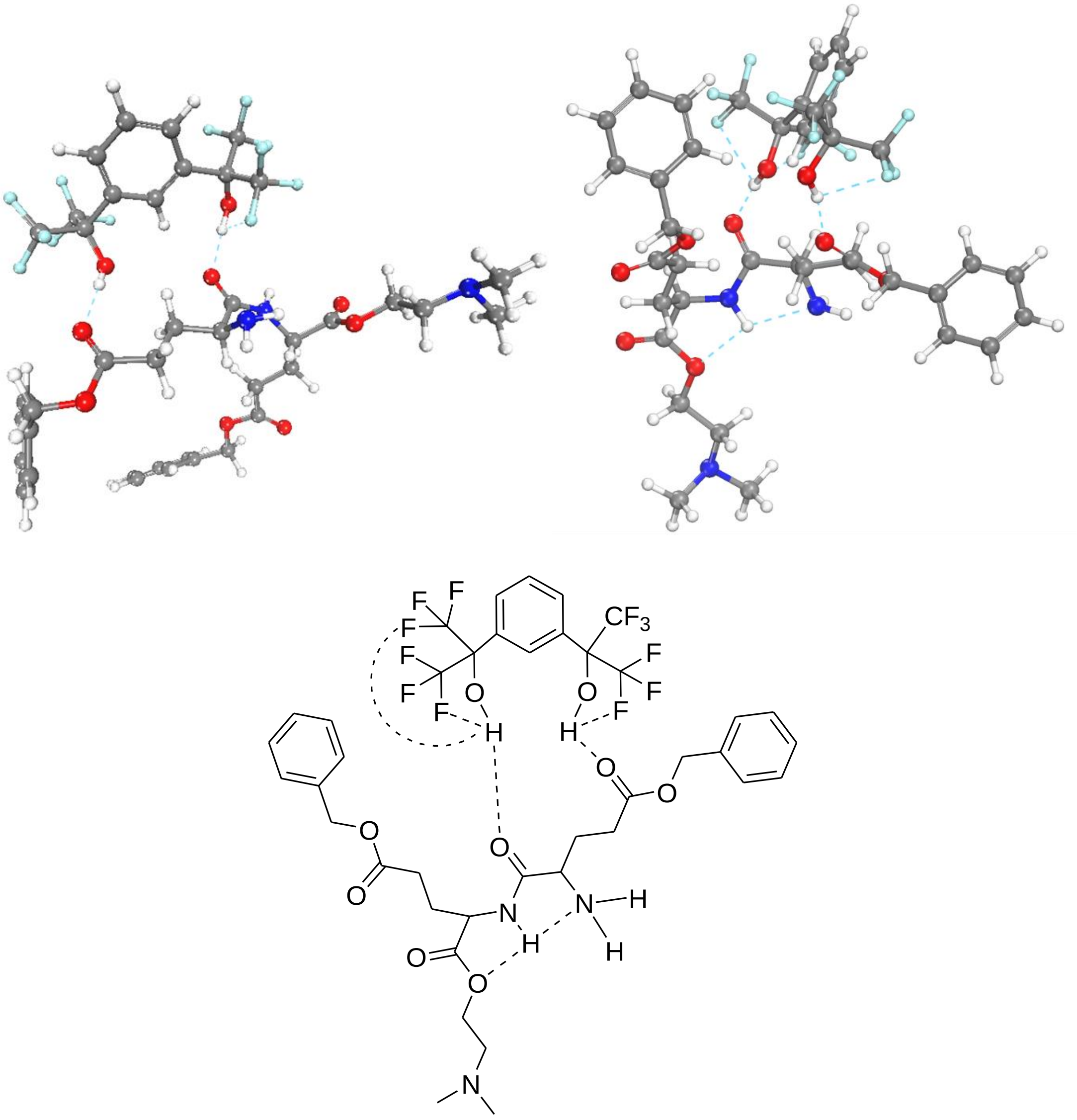

Catalyst-Initiator-Chain(MM)-11

Supplementary Figure 47 The configuration of catalyst-initiator-chain(MM)-11 complex in Supplementary Table 1 (chain(MM) represents two inserted monomer; on the left is the configuration of complex involving D-Glu-NCA)

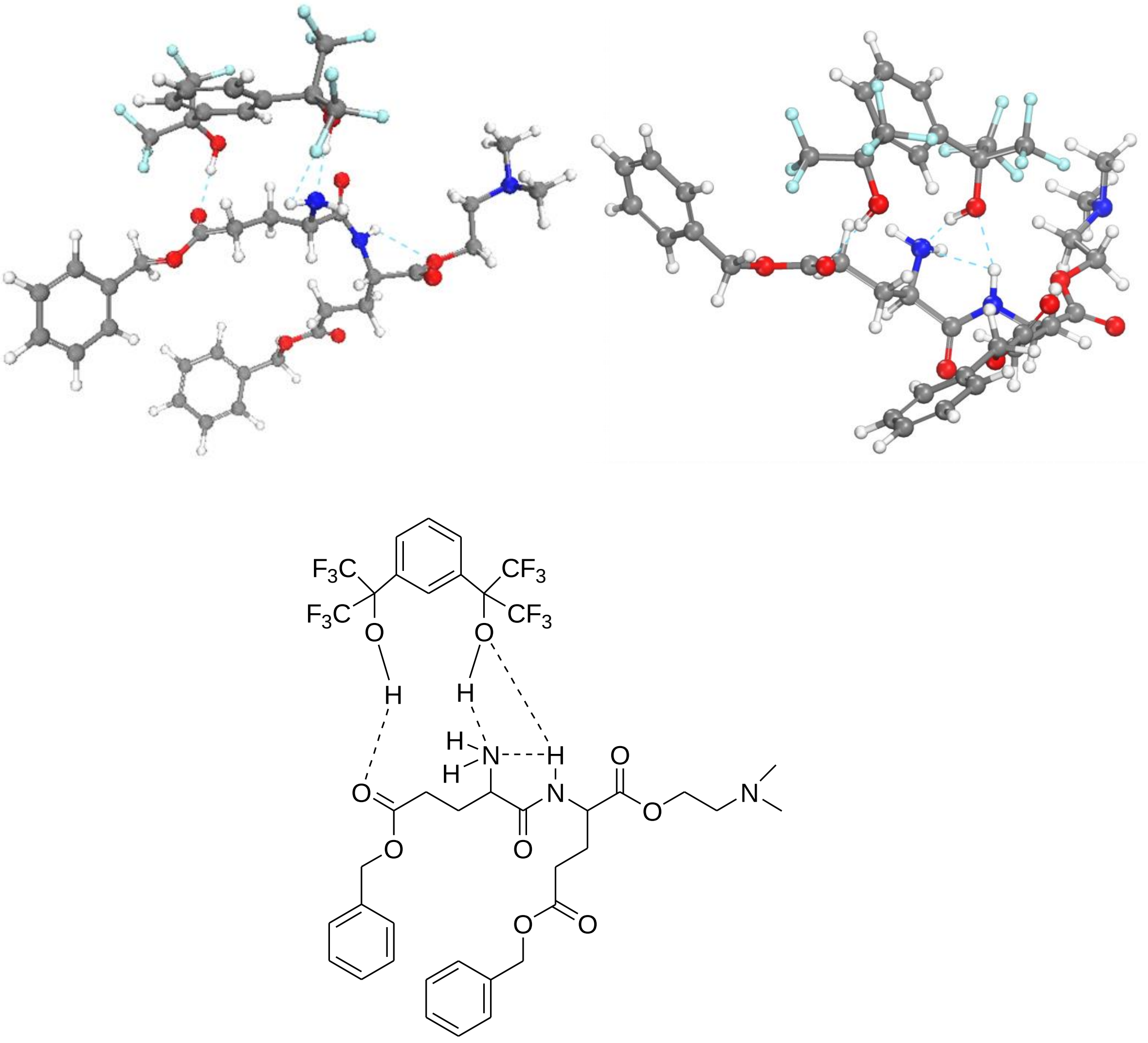

Catalyst-Initiator-Chain(MM)-12

Supplementary Figure 48 The configuration of catalyst-initiator-chain(MM)-12 complex in Supplementary Table 1 (chain(MM) represents two inserted monomer; on the left is the configuration of complex involving D-Glu-NCA)

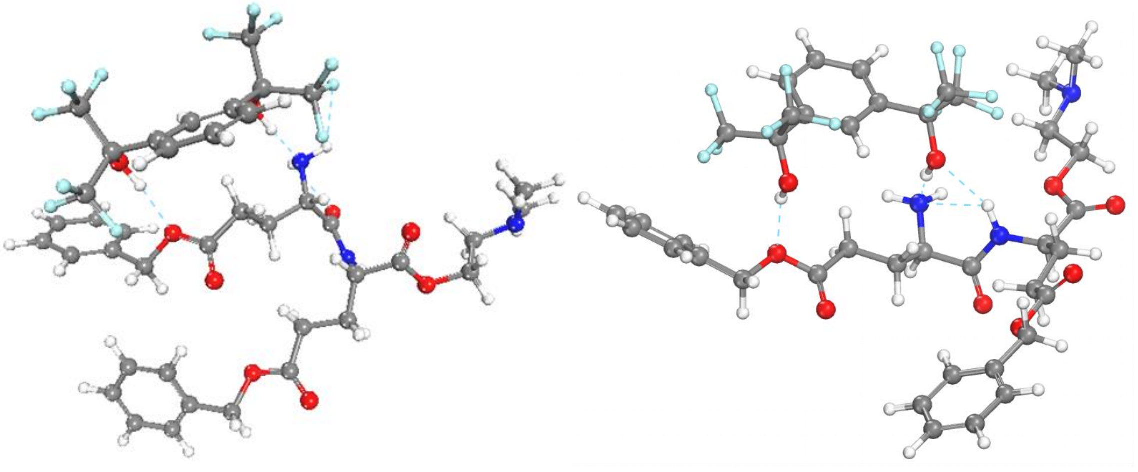

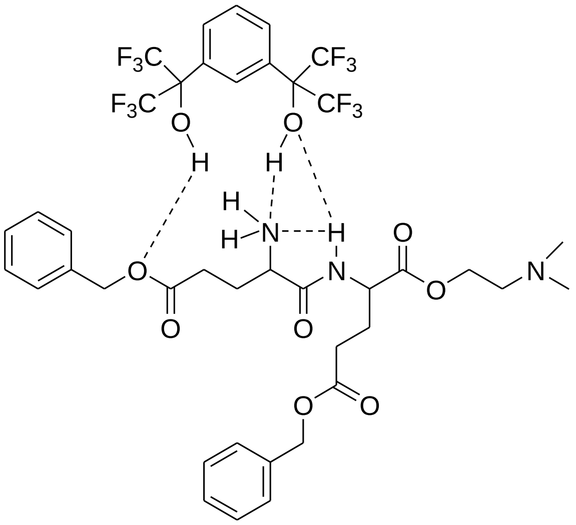

Catalyst-Initiator-Chain(MM)-13

Supplementary Figure 49 The configuration of catalyst-initiator-chain(MM)-13 complex in Supplementary Table 1 (chain(MM) represents two inserted monomer; on the left is the configuration of complex involving D-Glu-NCA)

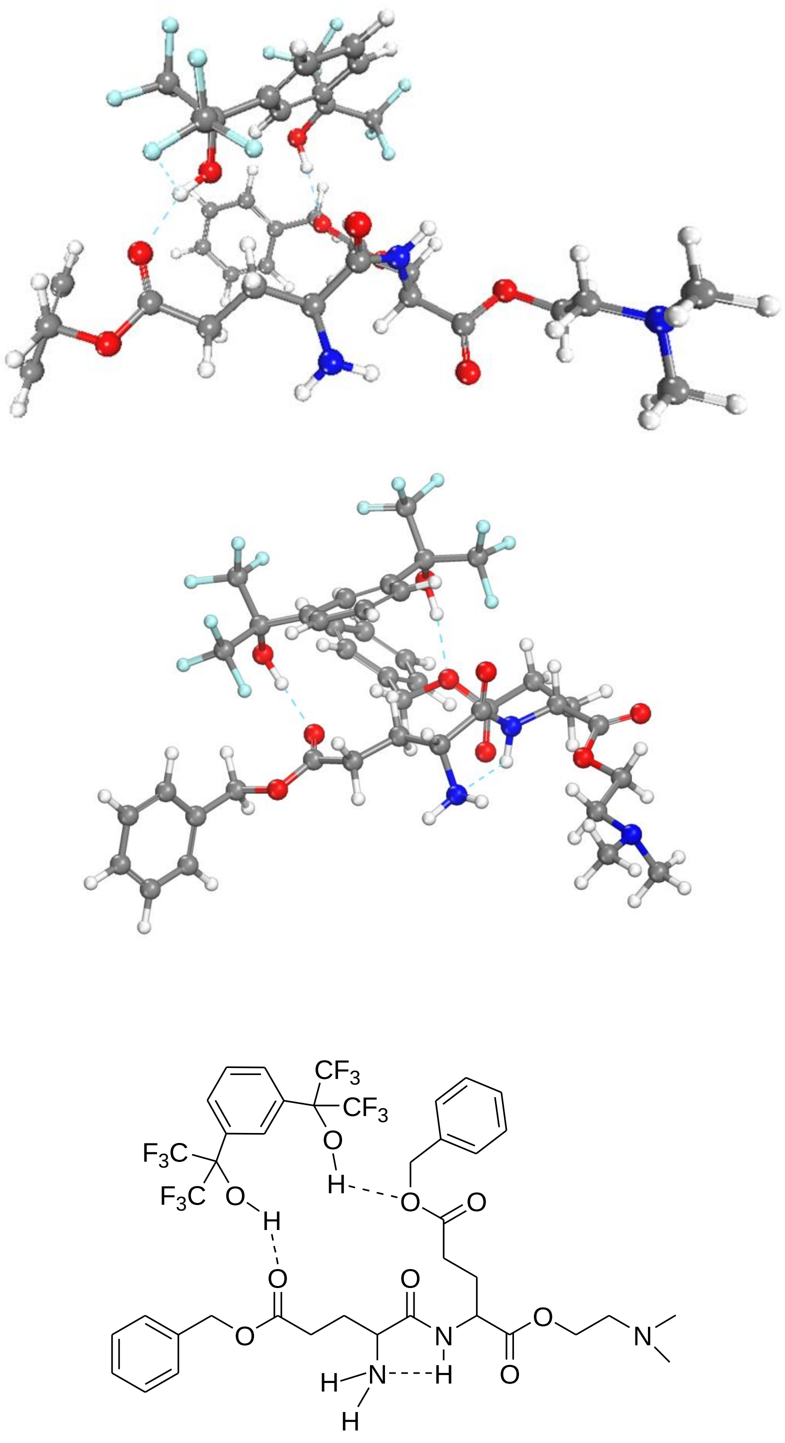

Catalyst-Initiator-Chain(MM)-14

Supplementary Figure 50 The configuration of catalyst-initiator-chain(MM)-14 complex in Supplementary Table 1 (chain(MM) represents two inserted monomer; below is the configuration of complex involving D-Glu-NCA)

Supplementary References

1. Lu, H. & Cheng, J. Hexamethyldisilazane-mediated controlled polymerization of alpha-amino acid *N*-carboxyanhydrides. *J. Am. Chem. Soc.* **129**, 14114-14115 (2007).

2. Engler, A., Lee, H., & Hammond, P. Highly efficient “grafting onto” a polypeptide backbone using click chemistry. *Angew. Chem. Int. Ed.* **48**, 9334-9338 (2009).

3. Zhu, L., Song, L., & Tong, R. Diastereoselective reductive ring expansion of spiroketal dihydropyranones to cis-fused bicyclic ethers. *Organic Letters* **14**, 5892-5895 (2012).

4. Hadjichristidis, N., Iatrou, H. Pitsikalis M. & Sakellariou, G. Synthesis of well-defined polypeptide-based materials via the ring-opening polymerization of *α*-amino acid *N*-carboxyanhydrides. *Chem. Rev.* **109**, 5528-5578 (2009).

5. Deming, T. J. Polypeptide and polypeptide hybrid copolymer synthesis via NCA polymerization. *Adv. Polym. Sci.* **202**, 1-18 (2006).
